# The Arabidopsis ABA-Activated Kinase OST1 Phosphorylates the bZIP Transcription Factor ABF3 and Creates a 14-3-3 Binding Site Involved in Its Turnover

**DOI:** 10.1371/journal.pone.0013935

**Published:** 2010-11-10

**Authors:** Caroline Sirichandra, Marlène Davanture, Benjamin E. Turk, Michel Zivy, Benoît Valot, Jeffrey Leung, Sylvain Merlot

**Affiliations:** 1 Institut des Sciences du Végétal (UPR 2355), CNRS, Gif-sur-Yvette, France; 2 UMR Génétique Végétale (UMR 0320/UMR 8120), Plate-Forme de Protéomique PAPPSO, INRA, Université Paris-Sud 11, CNRS, AgroParistTech, Gif-sur-Yvette, France; 3 IFR87 La plante et son environnement, Université Paris-Sud 11, INRA, CNRS, AgroParistTech, Gif-sur-Yvette, France; 4 Department of Pharmacology, Yale University School of Medicine, New Haven, Connecticut, United States of America; Purdue University, United States of America

## Abstract

**Background:**

Genetic evidence in *Arabidopsis thaliana* indicates that members of the Snf1-Related Kinases 2 family (SnRK2) are essential in mediating various stress-adaptive responses. Recent reports have indeed shown that one particular member, OPEN STOMATA (OST)1, whose kinase activity is stimulated by the stress hormone abscisic acid (ABA), is a direct target of negative regulation by the core ABA co-receptor complex composed of PYR/PYL/RCAR and clade A Protein Phosphatase 2C (PP2C) proteins.

**Methodology/Principal Findings:**

Here, the substrate preference of OST1 was interrogated at a genome-wide scale. We phosphorylated *in vitro* a bank of semi-degenerate peptides designed to assess the relative phosphorylation efficiency on a positionally fixed serine or threonine caused by systematic changes in the flanking amino acid sequence. Our results designate the ABA-responsive-element Binding Factor 3 (ABF3), which controls part of the ABA-regulated transcriptome, as a genuine OST1 substrate. Bimolecular Fluorescence Complementation experiments indicate that ABF3 interacts directly with OST1 in the nuclei of living plant cells. *In vitro*, OST1 phosphorylates ABF3 on multiple LXRXXpS/T preferred motifs including T451 located in the midst of a conserved 14-3-3 binding site. Using an antibody sensitive to the phosphorylated state of the preferred motif, we further show that ABF3 is phosphorylated on at least one such motif in response to ABA *in vivo* and that phospho-T451 is important for stabilization of ABF3.

**Conclusions/Significance:**

All together, our results suggest that OST1 phosphorylates ABF3 *in vivo* on T451 to create a 14-3-3 binding motif. In a wider physiological context, we propose that the long term responses to ABA that require sustained gene expression is, in part, mediated by the stabilization of ABFs driven by ABA-activated SnRK2s.

## Introduction

The plant hormone abscisic acid (ABA) regulates diverse aspects of plant growth and development including seed maturation, seed germination and root growth, and is a central component of biotic and abiotic stress responses, in particular, cold, salinity and drought [1]–[4]. In response to drought, ABA induces the closure of stomata to reduce water loss [5] and also reprograms gene expression leading to the accumulation of metabolites, sugars and Late Embryogenesis Abundant proteins (LEA) including dehydrins to protect cells from dehydration [6], [7].

In the past twenty years, numerous elements of ABA signaling have been identified, culminating with the recent establishment of a core ABA signaling pathway [8]. Based on genetic studies, it was previously established that clade A Ser/Thr Protein Phosphatase 2Cs (PP2C), including ABI1, ABI2 and HAB1 are major negative regulators of ABA signaling [9]–[15]. In contrast, the identification of the drought-sensitive Arabidopsis mutant *ost1*, also called *srk2e*, which does not close its stomata in response to ABA indicates that the ABA-activated Ser/Thr Snf1-Related Kinase 2 OST1/SRK2E/SnRK2.6 (hereafter called OST1), a homologue of *Vicia faba* AAPK, acts as positive regulator of ABA signaling in guard cells [16]–[18]. In addition to OST1, the Arabidopsis genome encodes two other SnRK2s strongly activated by ABA, SnRK2.2 and SnRK2.3 [19]. While single mutants are not distinguishable from the wild type, the double mutant *snrk2.2 snrk2.3* is insensitive to ABA inhibition of seed germination, root growth, and marker gene expression, but is not significantly affected in transpiration [20]. This genetic analysis indicated that SnRK2.2 and SnRK2.3 are redundant positive regulator of ABA signaling principally acting outside of guard cells.

A family of 13 START-domain containing proteins called PYR/PYL/RCAR were recently identified as the elusive soluble ABA receptors, which also bind to the catalytic site of the clade A PP2C leading to their inhibition [21]–[25]. In parallel, it was also shown that the clade A PP2Cs preferentially dephosphorylate a conserved Ser in the activation loop of ABA-activated SnRK2s leading to their inactivation [26]–[28]. In response to stress, such as drought, the binding of ABA to the PYR/PYL/RCAR/clade A PP2C complex releases the repression of ABA-activated SnRK2s to phosphorylate their substrates [24], [26]–[28]. Indeed, the *pyr1 pyl1 pyl2 pyl4* quadruple mutant, which displays broad ABA-insensitive phenotypes, is strongly compromised in the activation of SnRK2s by ABA [24], [29]. Although other ABA sensing pathway(s) may exist (for review [8]), the extreme ABA insensitivity of the triple mutant *ost1 snrk2.2 snrk2.3* indicates that protein phosphorylation mediated by ABA-activated SnRK2s is essential to regulate all aspects of ABA signaling [30]–[32].

In addition to phenotypic analysis, the identification of ABA-activated SnRK2s substrates will be critical to define the roles of these kinases in their physiological contexts. bZIP transcription factors of the ABA-responsive elements Binding Factor family (ABF), also called AREB, which regulate the transcription of ABA induced genes have been proposed to be genuine substrates of ABA-activated SnRK2s. ABFs bind to SnRK2s in yeast 2-hybrid and in plant cell [31], [33]. *In vitro,* SnRK2s phosphorylate several peptides containing RXX(S/T) motifs conserved in ABFs [20], [33]–[35], and the mutation of these sites affects ABF transcriptional activity in transient expression assays [34]. Using a combination of *in vitro* experiments and transient expression assay in plant cell protoplasts, it was recently shown that the PYR/PYL/RCAR proteins, clade A PP2Cs, ABA activated SnRK2s and ABFs constitute the core ABA signaling pathway leading to the transcription of ABA regulated genes [26]. The Arabidopsis *abf2(areb1) abf4(areb2) abf3* triple mutant is strongly affected in the induction of gene in response to ABA and very sensitive to drought stress [36]. However, the triple mutant is normal in transpiration suggesting that ABA-activated SnRK2s have additional substrate(s) in guard cells to regulate the closure of stomata. OST1 phosphorylates *in vitro* the potassium inward rectifying channel KAT1, the NADPH oxidase AtrbohF and the anionic channel SLAC1 [37]–[41]. These three plasma-membrane proteins are involved in the osmo-regulation of stomatal aperture [42]–[46]. However, the phosphorylation of these proteins by OST1 has not been demonstrated and analyzed *in vivo*. One further question central to ABA signaling is to understand how SnRK2-mediated phosphorylation regulates the activity of their substrates.

In this work, we have defined the OST1 phosphorylation site preferences using a combinatorial peptide array to predict OST1 targets at the genome scale in guard cells. Among the candidates, quantitative phosphorylation data from the peptides designates the transcription factor ABF3 as a likely physiological OST1 substrate. We confirmed this by showing that OST1 is able to phosphorylate *in vitro* ABF3 T451, located in a conserved LXRXXpTXP 14-3-3 binding motif. *In vivo,* T451 is essential for both ABA induced ABF3 phosphorylation and stability. This work thus suggests that during ABA signaling, one physiological role of SnRK2-mediated phosphorylation is to sustain the expression of a subset of ABA-regulated genes by slowing the degradation of specific ABFs.

## Results

### Determining OST1 motif preferences by phosphorylation of semi-degenerate peptides

**Figure 1:**
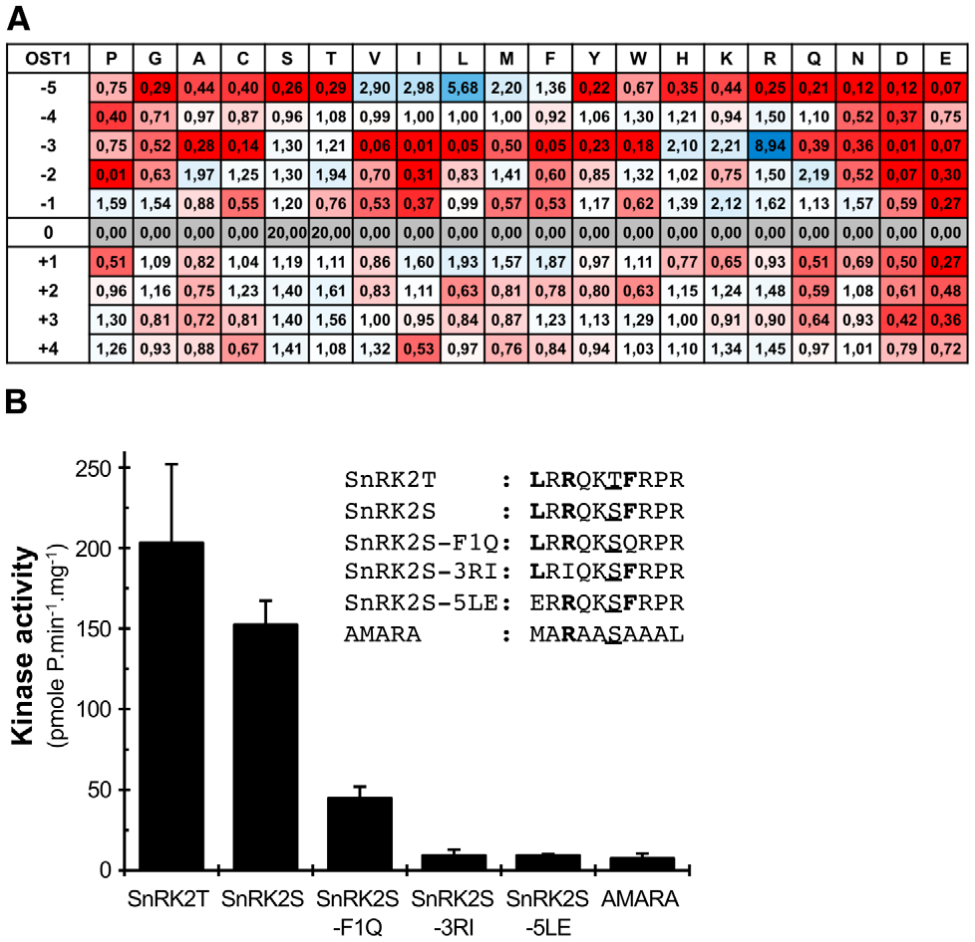
OST1 substrate preferences. (A) The Position Specific Scoring Matrix (PSSM) for the OST1 kinase regroups the calculated weight (W) of each amino acid at each position. The amino acids favoring phosphorylation (W>1) are colored in blue using Excel conditional formatting, while those that negatively affect phosphorylation (W<1) are in red. (B) A series of variant peptides originating from the optimized SnRK2S peptide was used to validate OST1 kinase phosphorylation preferences. The initial phosphorylation speed of the peptides (kinase activity) was calculated with a linear regression using 4 to 5 time-points of the phosphorylation kinetic. The error bars represent 95% confidence interval. The experiment was repeated tree times with similar results.

We experimentally defined the optimal phosphorylation motif of the kinase OST1 by screening a semi-degenerate combinatorial peptide array [47]. This approach measures the impact of each amino acid at the different positions (from −5 to +4) around the target site (position 0) on phosphorylation by a given kinase. We calculated the weight of each amino acid at each position as the ratio of the phosphorylation level of the corresponding peptide to the mean phosphorylation of the 20 peptides at the same position. These data were combined into a Position Specific Scoring Matrix (PSSM) to quantitatively define the motif preferences of OST1 (Figure 1A). These results revealed that OST1 has strong preferences for basic residue, principally Arg, at the −3 position and for a hydrophobic amino acid, mainly Leu, at the −5 position. In contrast, OST1 has a strong aversion for Pro at the -2 position. OST1 phosphorylates the SnRK2S and SnRK2T peptides, which combine residues favored by OST1 with a Ser or Thr as target site respectively, more efficiently than the AMARA peptide commonly used to measure the activity of Snf1 related kinases (Figure 1B). As predicted, the substitution of Arg at −3 by Ile (SnRK2S-3RI) and Leu at −5 by Glu (SnRK2S-5LE) reduces the phosphorylation of the corresponding peptides by more that 90%. The replacement of Phe by the hydrophilic Gln at the +1 position (SnRK2S-F1Q) leads to a 60% reduction of phosphorylation by OST1 supporting a moderate preference for hydrophobic amino acids (Leu, Phe, Ile, Met) at the +1 position. Together, these results indicate that OST1 preferentially phosphorylate the [LIVMF]_-5_X[RK]_-3_XX[S/T]_0_[LFIM]_+1_ motif. The preferences defined in this study are closely related to those of the osmotic stress-activated kinase SnRK2.10 [48], suggesting that SnRK2 kinases, at least *in vitro*, share very similar phosphorylation preferences.

### *In silico* prediction of OST1 substrates

We used the quantitative PSSM generated for OST1 to predict substrates using the MAST software (Table 1, Table S1). As OST1 has a predominant function in relaying ABA signaling to stomatal closure, our subsequent analysis on putative targets will be focused on those enriched in the guard cells (Table S2). This predictive analysis highlighted dehydrins, members of which have appeared consistently in ABA or drought-related transcriptomes. OST1 is predicted to phosphorylate the dehydrin RAB18 on S111 in its predicted optimal motif LXRXX(S/T) located at the N-terminal end of a series of serine residues called the S-segment. This was indeed confirmed by *in vitro* phosphorylation tests (Figure S1). It is evident, however, that the largest functionally coherent category of proteins, are the transcription factors of the ABF family. In particular, our *in silico* analysis revealed that the transcription factors ABF3 and ABF4 contain three optimal motifs conserved in ABFs (Table 1). Two sites corresponding to ABF3 S32 and S126 were previously shown to be phosphorylated in ABF2 and ABF4 by SnRK2 *in vitro* using short protein fragments [34]. In contrast, the third site corresponding to ABF3 T451 located in the conserved domain called C4 at the C-terminal extremity of ABFs had not been shown to be phosphorylated by SnRK2s [34]. The identification of several optimal OST1 phosphorylation sites in ABFs that are proposed to be physiological SnRK2 substrates indicates that this approach can be used to identify new substrates and also help to localize kinase specific phosphorylation sites on these proteins.

**Table 1:** Putative OST1 substrates identified in Arabidopsis annotated protein database using the MAST program.

Rank	E-value^a^	AGI	Description	Peptide	p-value^b^
*ABA-responsive element binding factors and transcription factors*					
18	11	At3g19290	bZIP ABRE Binding Factor ABF4 (AREB2)	LQRQGSLTLP	8,10E-07
	-			LRRTLTGPW*	3,10E-05
	-			LARQSSVYSL	4,40E-05
18	11	At1g45249	bZIP ABRE Binding Factor ABF2 (AREB1)	LQRQGSLTLP	8,70E-07
	-			LTRQGSIYSL	5,60E-06
21	12	At4g34000	bZIP ABRE Binding Factor ABF3 (DPBF5)	LQRQGSLTLP	8,80E-07
	-			LRRTLTGPW*	3,10E-05
	-			LTRQNSVFSL	3,10E-05
29	17	At3g56850	bZIP ABRE Binding Factor AREB3 (DPBF3)	LSRQGSLTLP	1,80E-06
	-			LNRQSSLYSL	4,10E-05
	-			LRRTSSAPF*	6,00E-05
29	17	At2g41070	bZIP Enhanced EM Level (DPBF4)	LVRQGSLTLP	2,10E-06
	-			LTRQNSLYSL	6,70E-06
	-			LRRTNSASL*	4,30E-05
33	18	At1g49720	bZIP ABRE Binding Factor ABF1	LQRQGSLTLP	1,50E-06
	-			LARQSSLYSL	8,80E-06
	-			LRRTLTGPW*	4,90E-05
	-			LERQQTLGEM	9,60E-05
41	23	At3g54320	Transcription factor WRINKLE 1	LRRQSSGFSR	2,10E-06
*Dehydrins*					
3	1,9	At1g20440	Dehydrin COR47	LHRSNSSSSS	2,40E-07
5	2,4	At1g20450	Dehydrin ERD10/LTI45	LHRSNSSSSS	3,00E-07
9	4,5	At5g66400	Dehydrin RAB18	LHRSGSGSSS	7,80E-07
16	8,9	At2g21490	Dehydrin protein	LRRSGSSSSS	1,60E-06
28	15	At3g50980	Dehydrin protein	LHRSGSSSSS	3,90E-06
*Glycine-rich proteins with unknown functions*					
1	1,6	At3g09070	Glycine-rich protein	LRRTKSFSAS	7,60E-08
5	2,4	At2g38070	Glycine-rich protein	LRRTKSFSAS	1,20E-07
8	4,3	At5g01170	Glycine-rich protein	LRRTKSFSAK	2,40E-07
*Metal binding proteins*					
4	2,3	At4g27590	Copper-binding protein	LIRTSSFTWK	4,80E-07
12	6	At2g02835	Zinc ion binding protein	LWRANTISIV	9,90E-07
15	8,8	At1g55915	Zinc ion binding protein	LSRQPSLSFL	6,90E-07
*Oligosaccharide metabolism*					
10	4,9	At4g19760	Glycosyl hydrolase family 18 protein	LSRAGSFSF*	4,30E-07
36	20	At4g19750	Glycosyl hydrolase protein	LSRAGSFSLT	1,80E-06
41	23	At3g22250	UDP-glucosyl transferase family protein	LERTKSLRWI	1,60E-06
*Translational control*					
24	13	At4g23620	50S ribosomal protein	LRRAKTLPKT	1,60E-06
41	23	At5g20290	40S ribosomal protein S8 (RPS8A)	LVRTKTLVKS	3,40E-06
41	23	At1g66890	Similar to 50S ribosomal protein-related	LRRRKTLRLL	4,90E-06
*Reversible protein phosphorylation*					
29	17	At4g08480	MAPKKK9	LLRQGSFGSV	7,10E-07
41	23	At5g66080	PP2C protein	LSRASSLKTP	1,90E-06
*Miscellaneous*					
7	4,1	At5g24880	Calmodulin-binding protein-related	LSRTKSLGRK	3,00E-07
	-			LLRRRSFDRP	1,10E-05
11	5,3	At1g76690	12-oxophytodienoate reductase OPR2	LTRQKSYGSV	4,50E-07
17	9,2	At5g59200	Pentatricopeptide repeat-containing protein	LSRRKTLISV	5,00E-07
18	11	At3g49150	F-box family protein	LRRTLSLRSL	5,80E-07
	-			LKRSLSSKTL	7,10E-05
21	12	At3g45243	ECA1 gametogenesis protein	LARAPSLTLA	3,50E-06
24	13	At5g10300	Hydrolase, alpha/beta fold protein	LHRQGSFFTE	1,60E-06
33	18	At5g19520	Ion channel domain-containing protein	LVRRKSLSRS	7,90E-07
39	21	At1g10070	Amino acid transaminase, ATBCAT-2	LSRAKSRGFS	1,90E-06
41	23	At1g04540	C2 domain-containing protein	LRRTKSDTSS	1,20E-06
*Unknown functions*					
2	1,8	At4g40020	Unknown protein	LVRRKSLSFS	9,20E-08
13	6,4	At2g31560	Unknown protein	LTRAKSLTDD	1,00E-06
14	8,1	At5g61710	Unknown protein	LRRRRTSNTR	1,70E-06
21	12	At4g26950	Unknown protein	LRRSRSSSSS	2,70E-06
24	13	At3g12870	Unknown protein	LRRAKSLRVE	2,00E-06
24	13	At2g43340	Unknown protein	LKRTKSLTDD	2,20E-06
29	17	At4g19970	Unknown protein	LTRSKSISFR	7,60E-07
33	18	At5g20900	Unknown protein	LNRAPSFSST	3,10E-06
36	20	At5g06280	Unknown protein	LRRTKSISNM	4,20E-06
36	20	At5g06280	Unknown protein	LRRTKSISNM	4,20E-06
39	21	At1g22110	Unknown protein	LSRTSSSSSS	2,30E-06
41	23	At3g27320	Unknown protein	LSRRNSLGSS	1,60E-06
48	24	At1g68330	Unknown protein	LRRSSSLSSS	2,90E-06

a The E-value of a sequence in a database is the expected number of sequences in a random database of the same size that would match the motif as well as the sequence does. Results are displayed for E-value ≤25.

b The position p-value is the probability of a single random subsequence of the length of the motif scoring at least as well as the observed match. Only peptides with position p-value ≤0.0001 are displayed.

### OST1 interacts with ABF3 in the nucleus of guard cells

**Figure 2:**
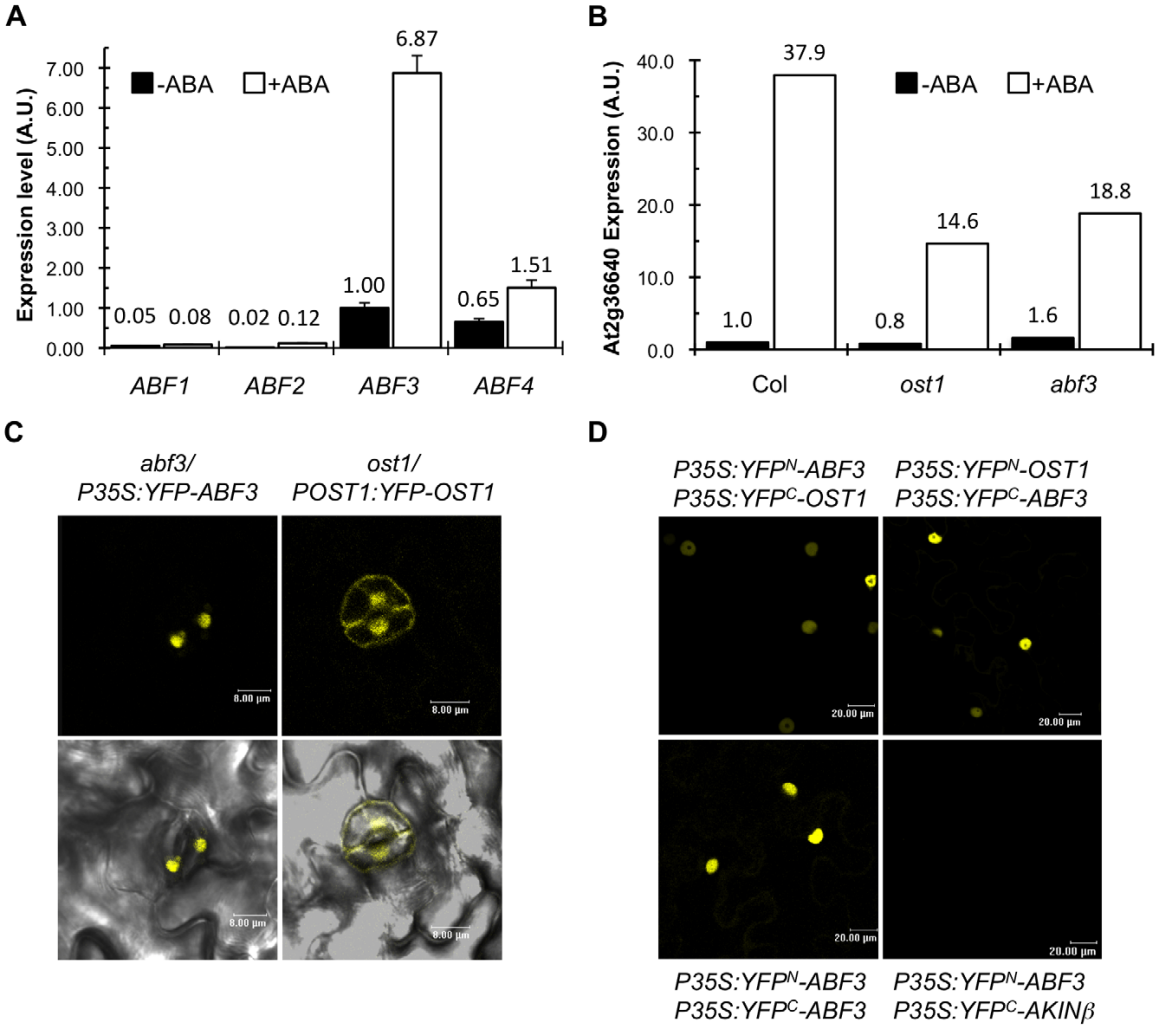
OST1 interacts with ABF3 in the nucleus of guard cell. (A) qRT-PCR analysis of *ABF* genes expression in epidermal peels in response to 50 µM ABA for 3 h. Data were normalized with *AtACTIN2* expression and the expression of *ABF3* gene without ABA was set to 1 as reference. Error bars indicate standard deviation for two technical replicates. A representative experiment out of three independent biological repetitions is shown (B) qRT-PCR analysis of *At2g36640* gene expression in epidermal peels of wild type (Col), *ost1* and *abf3* plant genotypes in response to 50 µM ABA for 3 h. *At2g36640* expression was normalized to the expression of *AtACTIN2* in all samples and the value corresponding to non-treated wild type plants (Col) set to 1. A representative experiment out of three independent biological repetitions is shown. (C) Confocal images of guard cells expressing YFP-ABF3 (left) and YFP-OST1 (right). On the bottom panel, the confocal images were merged with the corresponding bright field images. Scale bar corresponds to 8 µm. (D) ABF3 and OST1 interaction is analyzed by Bimolecular Fluorescent Complementation in *N. benthamiana* tobacco leaves expressing the given constructs. The YFP fluorescent signal is located in the nucleus of epidermal cells. In these experiments, the nuclear localized AKINβ protein is used as negative control. Scale bar corresponds to 20 µm.

Transcriptomic analysis indicated that ABF3 and ABF4 are expressed in guard cells [49]. To confirm these data, we used quantitative RT-PCR (qRT-PCR) to analyze the expression of ABF genes in epidermal peels containing functional guard cells (Figure 2A). We confirmed that *ABF3* and *ABF4* are significantly expressed in guard cell in contrast to *ABF1* and *ABF2*. In addition, *ABF3* expression is strongly induced by ABA, suggesting that ABF3 plays a major role in ABA signaling in guard cells. In agreement, we observed that the expression of the LEA gene *At2g36640* in response to ABA in guard cell is significantly reduced in Arabidopsis *abf3* mutant (Figure 2B). ABA induced expression of *At2g36640* is also affected in *ost1* mutant indicating that both OST1 and ABF3 are positive regulators of *At2g36640* expression in guard cell. We then analyzed the localization of YFP-OST1 and YFP-ABF3 fusion proteins that are able to complement the *ost1* and *abf3* mutants respectively (Figure S2). Both proteins are localized in nuclei but a fraction of YFP*-*OST1 is also present at the periphery of cells (Figure 2C). Bimolecular Fluorescence Complementation experiments using OST1 and ABF3 fused to either the N- and C-terminus of YFP further revealed that ABF3 interacts with OST1 in nuclei (Figure 2D). These data support the hypothesis that OST1 directly phosphorylates ABF3 in the nuclei of guard cells to activate the expression of genes in response to ABA.

### OST1 phosphorylates ABF3 on optimal sites *in vitro*

**Figure 3:**
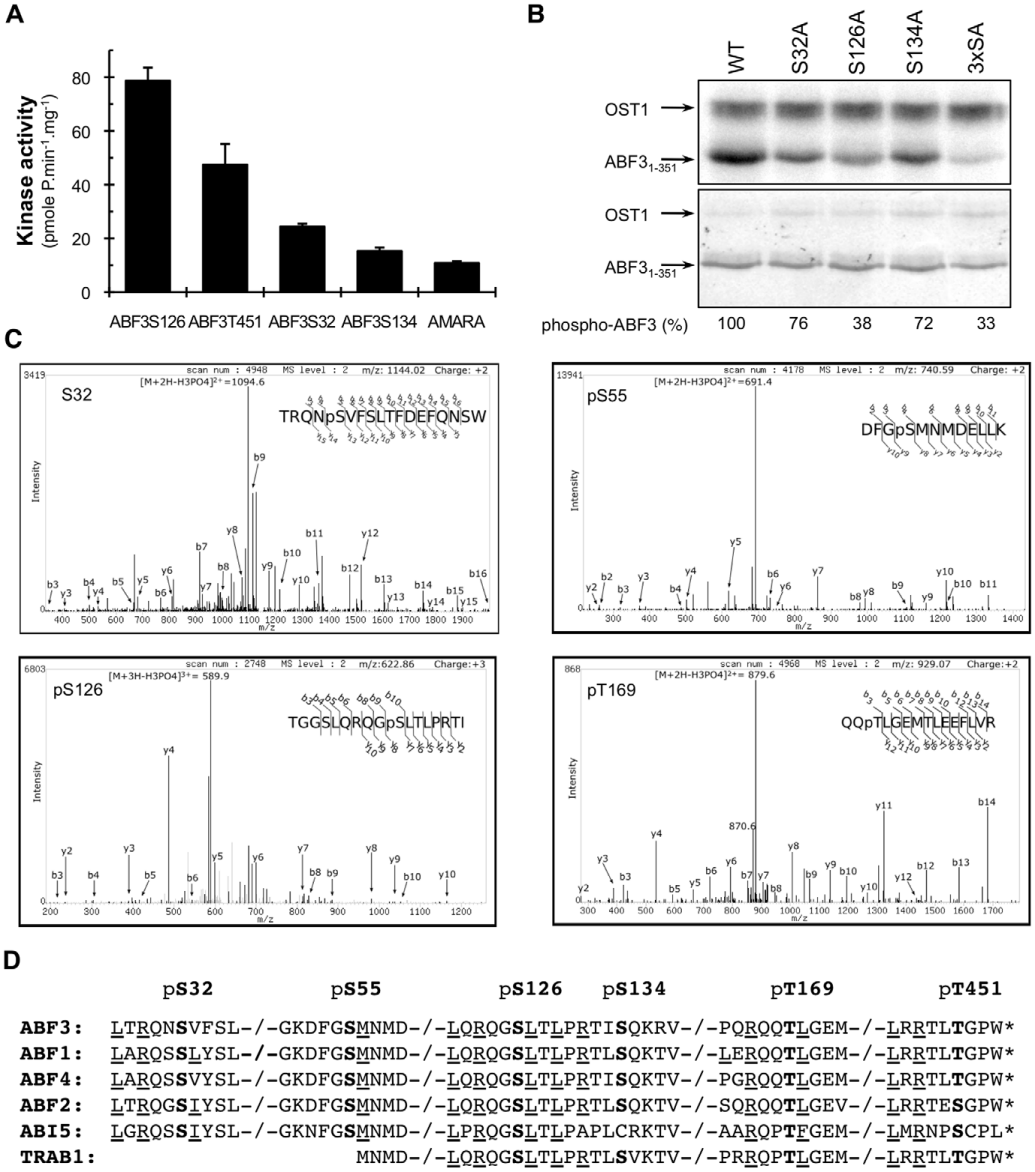
OST1 phosphorylates ABF3 on multiple sites *in vitro*. (A) OST1 kinase activity towards ABF3 derived peptides corresponding to the putative OST1 phosphorylation sites. The OST1 activity was calculated with a linear regression using 4 to 5 time-points of the phosphorylation kinetic. The error bars represent a 95% confidence interval. The experiment was repeated tree times with similar results. (B) The C-terminally truncated ABF3 protein (ABF31–351), and the corresponding mutant forms, in which target Ser were either individually or together (3xSA) mutated to Ala, were phosphorylated by OST1 *in vitro* in presence of ATP^γ32^ (upper panel). ABF3 phosphorylation was quantified by phosphorimaging and normalized to protein amount using Coomassie brilliant blue (CBB) gel staining (lower panel). ABF3 phosphorylation was set to 100% for the wild type protein. (C) ABF31–351 phosphorylation was analyzed by LC-MS/MS. Phosphorylation of S32, S126, S55 and T169 is revealed in MS2 spectra by the neutral loss of phosphoric acid group (H3PO4, 98 Da) from the corresponding precursor ion: the doubly charged TRQNpS32VFSLTFDEFQNSW ion at m/z 1144.02 was produced by chymotrypsin hydrolysis with miscleavages, the triply charged TGGSLQRQGpS126LTLPRTI ion at m/z 622.86 was produced by chymotrypsin hydrolysis, the doubly charged DFGpS55MNDELLK ion at m/z 740.59 and the doubly charged QQpT169LGEMTLEEFLVR ion at m/z 929.07 were produced by trypsin hydrolysis. (D) OST1 phosphorylation sites identified on ABF3 (At4g34000) were aligned with corresponding sequences in Arabidopsis ABF1 (At1g49720), ABF4 (At3g19290), ABF2 (At1g45249), ABI5 (At2g36270) and rice TRAB1 (Os08g0472000). Amino acids predicted to favor OST1 phosphorylation are underlined.

In addition to S32, S126 and T451, ABF3 S134 is also located in a conserved LXRXX(S/T) motif (Figure 3D), but was not highlighted as a putative OST1 target by the bioinformatic analysis because of its elevated position p-value. We measured the activity of OST1 towards these four putative target sites using peptides covering the surrounding sequences (Figure 3A). The highest OST1 activity was measured for ABF3S126 (82.1±5.3 pmol.min^−1^.µg^−1^) and ABF3T451 (47.9±3.4 pmol.min^−1^. µg^−1^) peptides corresponding to S126 and T451 respectively. OST1 also phosphorylates ABF3S32 (25.1±1.0 pmol.min^−1^. µg^−1^) and ABF3S134 (15.8±1.3 pmol.min^−1^. µg^−1^) with a higher activity than the AMARA peptide (10.3±1.3 pmol.min^−1^. µg^−1^). We attempted to confirm that OST1 phosphorylates the predicted sites in the context of the entire ABF3 protein. However, we were not able to purify the full-length His-tagged ABF3 using non-denaturating conditions because the protein was insoluble when expressed in bacteria. OST1 phosphorylates the truncated and soluble protein ABF3_1-351_ that covered most of protein but lacking the C-terminal bZIP and C4 domains (Figure 3B). Substitution of S32, S126 or S134 to Ala reduces ABF3_1–351_ phosphorylation by 24%, 62% and 28% respectively, but OST1 is still able to weakly phosphorylate the protein that combine these three mutations (abf3^3xSA^). These results indicated that *in vitro* OST1 phosphorylates ABF3_1-351_ mainly on the optimal LXRXX(S/T) motif S126, but also, with a lower activity, at least one suboptimal site.

### Mass spectrometry analysis reveals suboptimal OST1 phosphorylation sites on ABF3

To extend the identification of sites phosphorylated by OST1, we analyzed phosphorylated ABF3_1-351_ using liquid chromatography coupled to tandem mass spectrometry (LC-MS/MS). In this analysis, we covered 98% of ABF3_1-351_ protein and unambiguously identified phosphorylation on S32, S126 and S134 using neutral loss of the phosphate group (98 Da) during fragmentation of precursor ion (Figure 3C and Figure S3). This analysis also revealed the phosphorylation of T169 and S55 (Figure 3C). T169 is located in a RXXT motif conserved in ABFs. The corresponding T135 in ABF1 is located in the optimal LXRXXT motif that was predicted as a putative OST1 phosphorylation site (Figure 3D, Table 1). S55 is also located in a conserved motif among ABFs, but the presence of a negatively charged Asp at the -3 position was predicted to strongly impair phosphorylation by OST1. These results indicate that while OST1 preferentially phosphorylates LXRXX(S/T) motifs in ABF3 *in vitro,* it is also able to phosphorylate suboptimal sites.

### Phosphorylation of ABF3 T451 mediates interaction with 14-3-3 proteins *in vitro*

**Figure 4:**
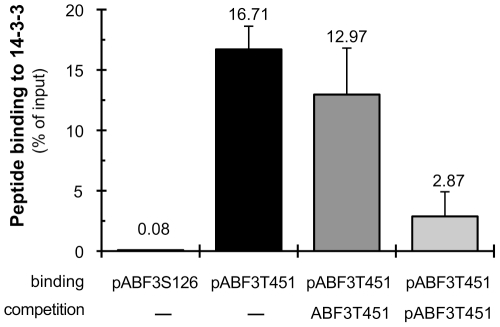
Phosphorylated C4 domain binds 14-3-3 protein. The binding of phosphorylated ABF3T451 (pABF3T451) and ABF3S126 (pABF3S126) peptides to the Arabidopsis 14-3-3 protein GF14phi was analyzed by *in vitro* pull-down assay and competition experiments. This experiment was repeated twice and error bar represent the standard error of the mean.

ABF3 T451 is located in a RXX(S/T)XP motif, conserved in ABFs including the seed specific ABI5 (Figure 3D), predicted to bind 14-3-3 proteins in a phosphorylation dependent manner [50]. This motif was shown to mediate the interaction of 14-3-3 proteins with ABFs from barley in yeast 2-hybrid [51]. Using pull-down assay, we showed that the ABF3T451 peptide phosphorylated by OST1 binds the Arabidopsis 14-3-3 protein GF14phi (Figure 4) contrary to ABF3S126 phosphorylated in the same condition. In addition, in competition experiments, we observed that the GF14phi-bound ABF3T451 peptide is displaced more efficiently by the phosphorylated ABF3T451 peptide than the non-phosphorylated peptide. These data show that the phosphorylation of T451 by OST1 increases the affinity of the ABF3 C4 domain for 14-3-3 proteins.

### ABF3 is phosphorylated on LXRXXS/T motif(s) in response to ABA in planta

**Figure 5:**
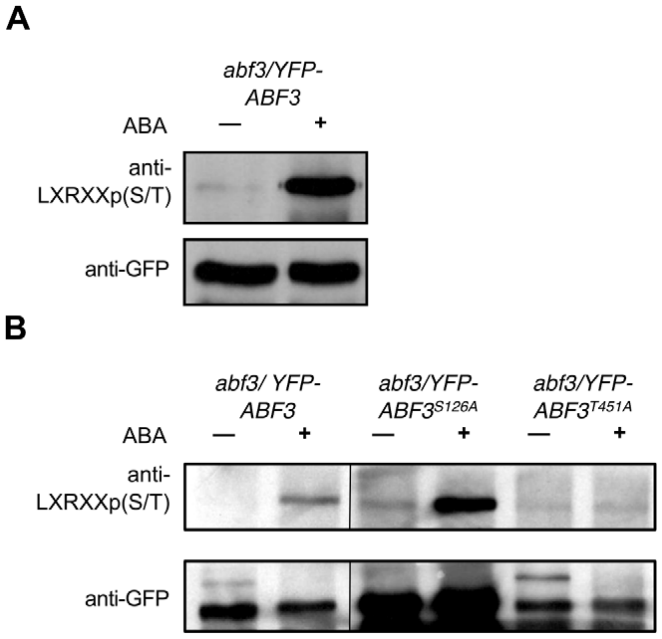
ABF3 is phosphorylated on LXRXX(S/T) motif(s) *in vivo* in response to ABA. Phosphorylation of immunoprecipitated YFP-ABF3 (A) and mutants YFP-abf3^S1261A^ and YFP-abf3^T451A^ (B) was analyzed by immunoblotting using the PKD substrate antibody that specifically recognized the phosphorylated LXRXXp(S/T) motif. In these experiments plants were treated with MG115 and MG132 for 24 h before treatment with 100 µM ABA for 1 h. The amount of immunoprecipitated proteins was controlled with anti-GFP antibody.

The OST1 optimal phosphorylation site LXRXX(S/T) is identical to the phosphorylation site of the mammalian PKD kinase [52]. We took advantage of the PKD substrate antibody that specifically recognizes the phosphorylated LXRXXp(S/T) motif to analyze ABF3 phosphorylation in transgenic plants expressing YFP-ABF3 (see next paragraph and material and methods). Using western blot analysis, we showed that YFP-ABF3 is recognized by the PKD substrate antibody only when plants are treated by ABA (Figure 5A). This result indicates that ABF3 is phosphorylated *in vivo* in response to ABA on at least one LXRXX(S/T) motif. We did not detect any phosphorylation of the mutant YFP-abf3^T451A^ in which T451 is mutated to Ala (Figure 5B), suggesting that ABF3 is predominantly phosphorylated on T451 in response to ABA. Alternatively, the primary phosphorylation of T451 might be required to prime the subsequent phosphorylation of other LXRXX(S/T) sites. In contrast, YFP-abf3^S126A^ is still significantly phosphorylated in response to ABA. While this indicates that the mutation of S126 does not have a major impact on the overall ABF3 phosphorylation *in vivo*, we cannot eliminate the possibility that this mutation may have incurred more subtle effects, difficult to detect because of the different expression levels of the variant YFP-ABF3 protein forms in transgenic plants.

### ABF3 T451 is critical for ABF3 stability

**Figure 6:**
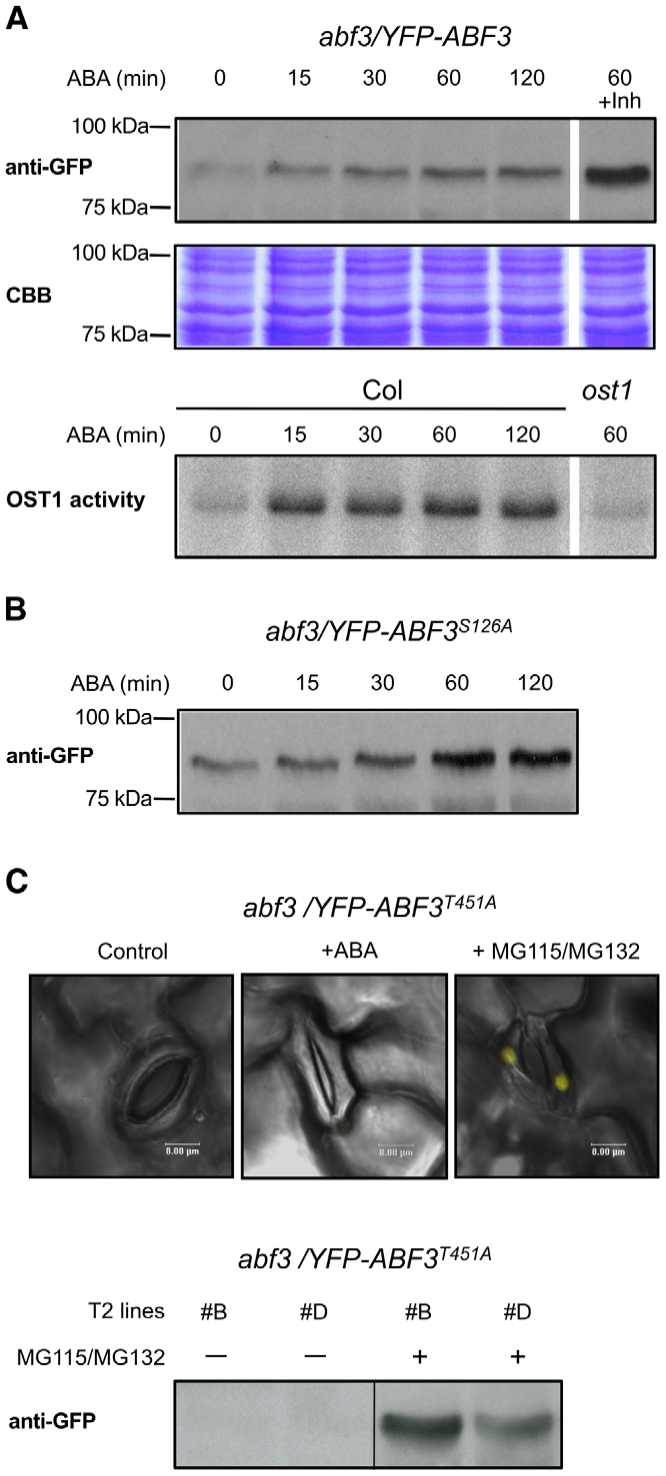
ABF3 is stabilized in response to ABA. (A) YFP-ABF3 stability in response to ABA was analyzed by immunoblotting with anti-GFP antibody using protein extract from transgenic plants expressing *YFP-ABF3* under the control of the constitutive 35S promoter (upper panel). Uniformity of protein loading was verified by CBB gel staining. This result is representative of three independent experiments. When indicated (+Inh), MG115 and MG132 proteasome inhibitors were added 1 h before ABA stimulation. The time course activation of OST1 by ABA was analyzed by kinase assay using 10xHis-ABF3_1-351_ as substrate (lower panel). The *ost1* knockout mutant is used as negative control in this experiment. (B) The effect of ABA on YFP-abf3^S126A^ stability was analyzed using the same experimental procedure. (C) The effect of ABA and proteasome inhibitors (24 h treatment) on YFP-abf3^T451A^ stability was analyzed by confocal microscopy (upper panel, merged fluorescent and bright field pictures, scale bar corresponds to 8 µm) using transgenic plants expressing *YFP-abf3^T451A^* under the control of the constitutive 35S promoter. This experiment was repeated independently twice with the same results. The stabilization of YFP-abf3^T451A^ by proteasome inhibitors was confirmed by immunoblotting (lower panel).

To analyze ABF3 *in planta*, we produced two polyclonal antibodies directed at ABF3-specific peptides and generated transgenic plants to express, under the control of the strong constitutive 35S promoter, ABF3 tagged N-terminally with either a triple HA epitope (3xHA-ABF3) or with the fluorescent YFP protein. In contrast to YFP-ABF3, we were not able to detect the native ABF3 or the tagged 3xHA-ABF3 proteins by western blot even after ABA treatment. We verified that 3xHA-ABF3 was expressed and functional in these transgenic lines by complementation of the *abf3* phenotype (Figure S2). These results suggested that only a low level of ABF3 is required in cells, and that the YFP fusion tag could have stabilized ABF3. YFP-ABF3 weakly but reproducibly accumulates 15 min after treatment with ABA and increase until 60 min (Figure 6A). The accumulation of YFP-ABF3 is also observed in transgenic plants treated with the proteasome inhibitors MG115 and MG132 (Figure 6A). The accumulation of YFP-ABF3 in response to ABA, which correlates with the rapid and prolonged activation of OST1 (Figure 6A), suggests that OST1 phosphorylation of ABF3 may regulate its turnover rate by the proteasome.

We then analyzed the stability of YFP-abf3^T451A^ in response to ABA. We generated 22 transgenic *abf3* lines expressing *YFP-abf3^T451A^* under the control of the strong constitutive 35S promoter, but we were not able to detect the YFP fusion protein in any of these lines using confocal microscopy or western blot analysis. As a comparison, we detected YFP-ABF3 and YFP-abf3^S126A^ in 4 out of 9 and 5 out of 16 transgenic lines respectively. We also observed that YFP-abf3^S126A^ is stabilized by ABA treatment indicating that S126 has no major implication in ABF3 stabilization (Figure 6B). We did not detected YFP-abf3^T451A^ after treatment of transgenic plants by ABA but the accumulation of YFP-abf3^T451A^ in the nucleus was restored by treatment with MG132 and MG115 (Figure 6C). These results suggest that T451 phosphorylation by ABA-activated kinases is required for stability of ABF3.

## Discussion

We have determined the substrate preferences of the ABA-activated kinase OST1 using a combinatorial peptide library screening strategy [47], [48]. We then exploited these quantitative data to identify putative targets in Arabidopsis protein databases. Among putative substrates identified in guard cells, we have analyzed in details the phosphorylation of ABF3 transcription factor. ABF3 interacts with OST1 in the nuclei of guard cells and both proteins positively regulate the expression of the LEA gene *At2g36640* suggesting that OST1 phosphorylation activates ABF3 in response to ABA. ABF3 contains four conserved SnRK2 optimal phosphorylation motifs LXRXX(S/T) that are phosphorylated by OST1 *in vitro* (Figure 3). The phosphorylation of ABF3 S32, S126 and S134 confirms previous studies showing that SnRK2s phosphorylate the corresponding motifs in other ABFs from Arabidopsis and rice *in vitro* [20], [34], [35]. In addition, we reveal that OST1 is also able to phosphorylate *in vitro* ABF3 T451 located in the conserved C4 LXRXXTXP motif at the C-terminal extremity of ABFs [34]. This C4 motif was recently shown to mediate the binding of 14-3-3 from barley with the ABF protein HvABI5 [51], [53]. Using pull-down analysis and competition experiments, we additionally show that OST1-mediated phosphorylation of ABF3 C4 domain on T451 increases its affinity for 14-3-3 proteins. It would be now important to test *in planta* if the phosphorylation of ABFs in response to ABA induces the interaction with 14-3-3s. The interaction between the phosphorylated C4 domain and 14-3-3 proteins also opens the possibility to design FRET-based sensors to study the spatiotemporal regulation of ABA-activated SnRK2s [54].

ABF transcription factors are phosphorylated in response to ABA *in vivo*, but the number and the identity of the phosphorylated sites were not clearly established [55]–[57]. Here, using the PKD substrate antibody that specifically recognize the phosphorylated LXRXXp(S/T) motif, we show that ABF3 is phosphorylated *in vivo* in response to ABA on at least one SnRK2 optimal phosphorylation site. Our results suggest that ABF3 is phosphorylated on T451 in response to ABA. Two recent quantitative phosphoproteomic studies also revealed that sites corresponding to ABF3 S126 and S55 are rapidly phosphorylated in response to ABA [58], [59]. These three sites are phosphorylated by OST1 *in vitro*, however we were not able to reveal a decrease of ABF3 phosphorylation in response to ABA in *ost1* plantlets probably because of the redundant activity of SnRK2.2 and SnRK2.3 (data not shown). The expression of *YFP-ABF3* in the recently isolated *ost1 snrk2.2 snrk2.3* triple mutant will be necessary to confirm and analyze the phosphorylation of ABF3 by ABA-activated SnRK2 *in planta* [30], [31]. In addition, other kinases including Calcium Dependant Protein Kinases, that share related phosphorylation preferences with SnRK2s, might participate in the phosphorylation of ABFs in response to ABA [48], [60], [61].

ABF3 is stabilized *in vivo* in response to ABA and also by inhibitors of the proteasome (Figure 6A), and this was also observed for the seed specific ABF ABI5 [56]. These results suggest that ABA-mediated stabilization of ABFs resulting from the inhibition of their degradation by the proteasome is a conserved mechanism implicated in ABFs activation. One important result of this study is that the substitution of ABF3 T451 to Ala strongly reduces ABA-mediated phosphorylation of ABF3 *in vivo* (Figure 5B) as well as its stability (Figure 6C). Therefore, these results establish a link between the phosphorylation of ABFs by ABA-activated SnRK2s on the C4 domain and their stabilization, and further suggest that 14-3-3s protect ABFs from rapid turnover. This role of 14-3-3s in ABA signaling is supported by the recent observation that in barley 14-3-3s positively regulate the activity of the ABF HvABI5 [53]. 14-3-3s may act in concert with the RING E3 ligase KEEP ON GOING [62] and ABI Five Binding Proteins [63], [64] that have been previously shown to regulate ABI5 turnover. The phosphorylation of the C4 domain is unlikely the only mechanism implicated in ABF activation by ABA since transient transactivation assays in Arabidopsis cells indicated that the full activation of ABF2 requires phosphorylation on multiple sites targeted by SnRK2s [34]. Bringing these data together, we propose that ABA-activated SnRK2s phosphorylate ABFs stepwise, first on the C4 domain leading to their accumulation, then on other sites to complete their activation to induce the transcription of ABA regulated gene.

Our bioinformatics screen identifies other putative OST1 substrates in stomata including dehydrins and the glutathione peroxidase AtGPX3 (Table S2). Arabidopsis at*gpx3* mutants are impaired in stomatal closure in response to ABA [65], suggesting that the phosphorylation of AtGPX3 by OST1 may be involved in the regulation of stomatal closure. In addition, we have shown that the NADPH oxidase AtrhohF, acting downstream of OST1 in ABA signaling [17], [42], is phosphorylated on LXRXXp(S174) by OST1 *in vitro* [40]. OST1 also phosphorylates the anionic channel SLAC1 *in vitro* on FSRQVp(S59)L and LSKQKp(S120)L motifs corresponding to motifs preferentially phosphorylated by OST1 [37], [41]. The substitution of S120 to Ala strongly affects the activity of SLAC1 suggesting that phosphorylation of S120 by OST1 is important to activate SLAC1 [37], [41]. We therefore think that OST1 phosphorylation preferences can be used to identify new physiological substrates of ABA-activated SnRK2s. However, this strategy might be limited by the fact that the screening of the combinatorial peptide library, representing 10^12^ individual motifs, only reveals optimal phosphorylation sites. For example, OST1 phosphorylates the potassium channel KAT1 on T306 that is not located in a favored phosphorylation site [39]. In the present study, the phosphorylation of ABF3 on GKDFGp(S55)M motif was particularly surprising because Asp at the -3 position is predicted to strongly disfavor phosphorylation by OST1 (Figure 1A). It was recently shown that SnRK2.8 autophosphorylates *in vitro* on the VKDIGpS motif and phosphorylates two 14-3-3s on IRDYRpS and VKDYRpS motifs respectively [66]. These results suggest that the (K/R)DXXS motif represent an unrelated SnRK2 phosphorylation site that was not revealed by the screening of the combinatorial peptide library. Alternatively, as shown for mammalian AMPK and cauliflower HMG-CoA reductase kinase [67], a basic residue at the -4 position in SnRK2 substrates may compensate for a lack of Arg at the -3 position. Additional studies will be required to demonstrate that OST1 can phosphorylate suboptimal sites *in vivo.*

Although the scanning of combinatorial peptides may miss the less-preferred phosphorylation sites, those corresponding to the *in vitro* definition of “optimal” will help to uncover the panoply of possible candidate substrates (Table 1). The discovery of these new ABA-activated SnRK2 substrates and the understanding of their regulation by phosphorylation will contribute to a more coherent understanding of ABA and drought stress signaling in diverse physiological contexts, beyond the description of their individual biochemical activities.

## Materials and Methods

### Plant mutants, culture conditions and phenotypic analysis

The Arabidopsis *ost1* knockout mutant corresponding to the *srk2e mutant* [18], was kindly provided by Dr. Kazuo Shinozaki. The *abf3* knockout mutant was isolated from the T-DNA line SALK_075836 obtained from NASC. These mutants are in the *Arabidopsis thaliana* Columbia accession (Col). Plants were routinely grown in a greenhouse (22°C, 16 h light), but plants used for epidermal peel preparation were grown in a culture cabinet (60% humidity, 8 h light, 22°C/18°C light/dark cycles). For root growth phenotype analysis, surface-sterilized seeds were germinated on 0.5X Murashige and Skoog (MS) agar medium supplemented with 1% sucrose (21°C, 16 h light). Two to three days old seedlings were transferred to the same medium containing 30 µM ABA and plates were incubated vertically for 5 days before measuring root growth. Leaf temperature was analyzed as previously described [13].

### Identification of OST1 substrate preferences and putative substrates

OST1 substrate preferences were determined by screening a semi-degenerate peptide library as previously described [47] using the active OST1 kinase expressed in *E. coli* [68]. OST1 substrate preferences data were compiled into a Position Specific Scoring Matrix (PSSM) and used with MAST program to scan the *Arabidopsis thaliana* annotated protein database TAIR version 7 [48], or proteins encoded by the 5000 most strongly expressed genes in guard cells [49].

### OST1 kinase activity against peptides and proteins

OST1 kinase activity was measured essentially as previously described [48] using the following peptides: AMARA, AMARAASAAALARRR; SnRK2S, YALRRQKSFRPRKKK; SnRK2T, YALRRQKTFRPRKKK; SnRK2S-5LE, YAERRQKSFRPRKKK; SnRK2S-3RI, YALRIQKSFRPRKKK; SnRK2S-F1Q, YALRRQKSQRPRKKK; ABF3S32, MALTRQNSVFSLKKK; ABF3S126, MALQRQGSLTLPKKK; ABF3S134, MALPRTISQKRVKKK and ABF3T451, KKRQCLRRTLTGPW. Peptides (250-500 µM) were phosphorylated in 50 µl reaction containing kinase buffer (20 mM HEPES pH 7.4, 20 mM MgCl_2_, 1 mM DTT, 25 mM β-glycerophosphate), 100 µM of ATP^γ32^ (0.05–0.09 µCi. nmol^−1^) and purified OST1 (100–500 ng).

The *ABF3* coding sequence from amino acids 1 to 351 was amplified from full-length cDNA clone pda14224 (Riken Bioresource Center) using primers ABF3_F and ABF3_R (See supporting Text S1 for primer sequences). S32, S126 and S134 were mutated to Ala using the mutagenic primers ABF3S32_F, ABF3S32_R, ABF3S126_F, ABF3S126_R, ABF3S134_F, ABF3S134_R. The PCR products were cloned into the XhoI site of pET-16B vector (Novagen) and construct verified by sequencing. The resulting fusion proteins (10xHis-)ABF3_1-351_, abf3^S32A^, abf3^S126A^, abf3^S134A^ and abf3^3xSA^, in which the 3 Ser are mutated, were expressed and purified from *E. coli* according to standard protocol. ABF3 proteins (2 µg) were phosphorylated by OST1 (1 µg) in 60 µl reaction containing the kinase buffer and 100 µM ATP^γ32^ (0.75 µCi.nmol^−1^ ATP). The reaction was stopped after 30 min by the addition of 6X Laemmli solution and proteins resolved in 10% SDS-PAGE. Gels were stained with Coomassie blue, dried and the radioactive signal quantified by phosphorimaging.

*In planta* OST1 kinase activation by ABA was measured as previously described [68] using protein immunoprecipitated from whole plant extract and 2 µg of 10xHis-ABF3_1-351_ as substrate.

### 14-3-3 pull-down assay

The *Arabidopsis* 14-3-3 protein GF14phi fused to GST was expressed in *E. coli* using a construct kindly provided by Dr. Ken-ichiro Shimazaki [69]. ABF3T451 and ABF3S126 peptides were phosphorylated by OST1 in presence of 100 µM ATP^γ32^ (0.05 µCi.nmol^−1^ATP) and then incubated (15 µM peptide) for 30 min at 25°C with 20 µl of 50% slurry glutathione beads loaded with 0.4 nmole of GST-14-3-3 in binding buffer (20 mM Tris-HCl pH 7.5, 150 mM NaCl, 1 mM CaCl_2_, 1 mM DTT, 0.05% Tween20) to a final volume of 200 µl. Beads were extensively washed with binding buffer before quantification of bounded peptide by Cerenkov counting. For competition experiments, beads were incubated for 30 minutes at 25°C in 200 µl binding buffer containing 100 µM of either ABF3T451 or ABF3T451 phosphorylated by OST1 with cold ATP. Beads were separated from supernatant by centrifugation and the radiolabeled phospho-ABF3T451 quantified in each fraction.

### Identification of phosphorylation sites by LC-MS/MS analysis

10xHis-ABF3_1-351_ phosphorylated by OST1 was digested in-gel with modified trypsin (Promega) or chymotrypsin (Sigma) using the ProGest system (Genomic Solutions). Peptides were separated on an Ultimate LC system (PepMap C18 column) combined with a Famos autosampler and a Switchos II microcolumn switch system (Dionex Corp.). Eluted peptides were analyzed on-line with a LTQ XL ion trap (Thermo Electron Corp.) using a nano-electrospray interface, and peptide ions were analyzed using Xcalibur 2.07. Database search was performed with Bioworks 3.3.1 (Thermo Electron Corp.) using a personal database containing ABF3, keratins and protease sequences. Phosphorylated peptide were identified in MS2 scan by neutral loss (98 Da) of precursor ion. The detailed LC-MS/MS procedure is described in supporting Text S1.

### Quantitative analysis of gene expression

Total RNAs were extracted from epidermal fragments enriched in guard cell prepared essentially as described [70]. DNA-free RNAs were purified using the RNeasy plant mini kit according to the manufacturer's instructions (Qiagen). First-strand cDNA was produced using the Superscript III first-strand synthesis system (Invitrogen). The sequences of the primers used in quantitative RT-PCR experiments (qRT-PCR) to amplify *ABF1* (At1g49720), *ABF2* (At1g45249), *ABF3* (At4g34000), *ABF4* (At3g19290), the *LEA* gene At2g36640 [49], *ACTIN2* (At3g18780) and *TIP41-like* gene (At4g34270) are given in Supplementary experimental procedures. qRT-PCR reactions were performed using the LightCycler FastStart DNA Masterplus SYBR Green I kit (Roche). For gene expression analysis at least two independent biological replicates and two technical replicates using two independent cDNA synthesis from the same RNA sample were used. Gene expression was normalized to *ACTIN2* and *TIP41-like* gene expression used as constitutive controls. More details are given in supporting Text S1.

### Expression of YFP fusion proteins in transgenic plants

Full length *ABF3* coding sequence was amplified by PCR using *att*B1ABF3_F and *att*B2ABF3_R primers. T451 to Ala mutation was introduced using the *att*B2ABF3TA_R primer and S126A mutation was obtained as described in the previous section. The PCR products were first introduced in pDONR221 and then pEarleyGate104 [71] using Gateway cloning (Invitrogen) to express YFP-ABF3, YFP-abf3^S126A^ and YFP-abf3^T451A^ under the control of the 35S promoter in plants.

To express YFP-OST1 under the control of the OST1 promoter region in transgenic plants, the OST1 promoter region was isolated as a EcoRI fragment from *POST1:GUS* construct [17] and cloned into pCambia1390. The *OST1* coding sequence was amplified from cDNA with *att*B1OST1_F and *att*B2OST1_R and cloned in pEarleygate104. The *YFP-OST1* fusion was subsequently amplified with the NcoIYFP_F and NcoIOST1_R and cloned as a NcoI fragment downstream of the *OST1* promoter in pCambia1390.

These constructs were transformed in *abf3* or *ost1* mutants by *Agrobacterium tumefaciens* transformation [72]. Homozygous T2 plant lines were selected for further experiments.

### BiFC experiments and confocal imaging analysis

*OST1* and *ABF3* coding sequences were recombined by Gateway reaction into pYFP^N^43 and pYFP^C^43 vectors (kindly provided by A. Ferrando, University of Valencia, Spain, http://www.ibmcp.upv.es/FerrandoLabVectors.php) that are derivatives of pMDC43 [73]. The *pYFP^N^43-*AKINβ construct (provided by A. Ferrando), to express YFP^N^-AKINβ, was used as a negative control. The corresponding YFP^N^ and YFP^C^ fusion proteins were transiently expressed in *Nicotiana benthamiana* leaves essentially as described [74]. Bimolecular Fluorescence Complementation was examined 3 days after leaf infiltration using a Leica Sp2 inverted confocal microscope with YFP settings (Excitation 488 nm, Emission 535–580 nm). The same microscope settings were used to analyze YFP-ABF3 and YFP-OST1 cellular localization in Arabidopsis transgenic plants.

### Immunological analysis of protein accumulation and phosphorylation

Proteins were extracted from thirty 3-weeks-old *in vitro* transgenic plants treated by ABA (100 µM) in 0.5X MS liquid medium and then frozen. When mentioned, plants were treated for 1 h with proteasome inhibitors MG115 (10 µM) and MG132 (25 µM) before ABA treatment. Plants were grinded using a Retsch Mill MM 301 and proteins extracted in lysis buffer [50 mM Tris pH 7.4, 150 mM NaCl, 10 mM NaF, 25 mM β-glycerophosphate, 3 mM Na-pyrophosphate, 1 mM EDTA, 1 mM EGTA, 10% (v/v) glycerol, 1% Triton-X-100, 5 mM DTT], containing EDTA-free protease inhibitors (Roche, 11836170001) and phosphatase inhibitors (Sigma, P5726). The crude lysate was cleared by centrifugation and soluble proteins concentration determined using Bradford assay (Thermo Scientific, 1856209). For the analysis of proteins phosphorylation, YFP tagged proteins were immunoprecipitated with 4 µg of mouse anti-GFP antibody (Roche, clones 7.1 and 13.1) from 4.5 mg soluble proteins for 90 min at 4°C in a final volume of 1 ml. Immunocomplexes were purified by incubation with 50 µl of 50% slurry Dynabeads® Protein G (Invitrogen, 100.03D) for 30 min followed by extensive washes with lysis buffer. Soluble proteins and immunocomplexes were analyzed by immunoblotting using chemiluminescence ECL plus reagent (GE Healthcare). YFP tagged proteins were analyzed using mouse anti-GFP antibody. Protein phosphorylation was analyzed using the PKD substrate antibody exactly as recommended by the supplier (Cell signaling, 4381).

## Supporting Information

Text S1 Supplementary methods.
(0.06 MB DOC)

Table S1 (0.14 MB DOC)

Table S2 (0.11 MB DOC)

Figure S1 OST1 phosphorylates RAB18 *in vitro*. (10xHis-)RAB18 and the Arg108 to Ala mutant (10xHis-)rab18^R108A^ was phosphorylated by OST1 in vitro. Protein were analyzed in a SDS-PAGE gel containing Mn^2+^-Phos-tag and stained with Coomassie blue. Phos-tag specifically chelates phosphate group and slow down the migration of phosphorylated protein [75].
(0.17 MB TIF)

Figure S2 Complementation of *abf3* and *ost1* mutants by expression of fusion proteins. (A) The root growth of Arabidopsis WT (Col), *abf3* mutant and *abf3* transgenic line expressing *YFP-ABF3* under the control of the *35S* promoter was measured in absence and presence of 30 µM ABA [76]. (B) The root growth of Arabidopsis WT (Col), *abf3* and *abf3* transgenic line expressing *[3xHA]-ABF3* under the control of the *35S* promoter was measured in presence of 30 µM ABA. (C) Detached leaf temperature analysis of Arabidopsis WT (Col), *ost1* (*srk2e*) and *ost1* transgenic line expressing *YFP-OST1* under the control of the *OST1* promoter. In these analyses, error bars represent the standard deviation of the mean.
(0.09 MB TIF)

Figure S3 Identification of phosphorylated ABF3 S134 by LC-MS/MS. Phosphorylation of S134 is revealed in MS2 spectra by the neutral loss of phosphoric acid group (H_3_PO_4_, 98 Da) from the triply charged precursor ion TIpS_134_QKRVDDVWK ion at m/z 519.00 produced by trypsin hydrolysis with miscleavages. The fragmentation of this peptide was not annotated by the Bioworks 3.3.1 program.
(0.03 MB TIF)
