# Supplementary material for: The Arabidopsis ABA-Activated Kinase OST1 Phosphorylates the bZIP Transcription Factor ABF3 and Creates a 14-3-3 Binding Site Involved in Its Turnover
Source: PLoS One. 2010 Nov 10;5(11):e13935. doi: 10.1371/journal.pone.0013935 (PMC2978106; doi:10.1371/journal.pone.0013935)
Supplement: Text S1 — Supplementary methods. (0.06 MB DOC) [file pone.0013935.s001.doc]

**Supplementary experimental procedures**

***Identification of phosphorylation sites by LC-MS/MS analysis***

10xHis-ABF3_1‑351_ was phosphorylated by OST1 for 4 h with cold ATP. Proteins were separated in a SDS-PAGE gel, and in-gel digestion was performed with the ProGest system according to the standard trypsin protocol (Genomic Solutions, MI). Gel pieces were washed twice by successive baths of 10% acetic acid, 40% ethanol, 50% acetonitrile (ACN), and then washed twice with successive baths of 25 mM NH_4_HCO_3_ and ACN. Cysteines were reduced in 10 mM DTT, 25 mM NH_4_HCO_3_ at 55°C for 30 min and then alkylated in presence of 50 mM iodoacetamide in 25 mM NH_4_HCO_3_ at 20ºC for 45 min. Digestion was performed for 6 h at 37°C with 125 ng of modiﬁed trypsin (Promega), or chymotrypsin (Sigma) dissolved in 20% methanol and 20 mM NH_4_HCO_3_. Peptides were extracted successively with 2% triﬂuoroacetic acid (TFA), 50% ACN and then with 100% ACN. Peptides were dried and suspended in 20 µL of 0.05% TFA, 0.05% HCOOH, 2% ACN.

HPLC was performed on an Ultimate LC system combined with a Famos autosampler and a Switchos II microcolumn switch system (Dionex Corp., CA). A 4 µL sample was loaded at 5 µL/min on a precolumn cartridge (stationary phase: C18 PepMap 100, 5 µm; column: 300 µm i.d., 5 mm; Dionex Corp.) and desalted with 0.05% TFA, 0.05% HCOOH, and 2% ACN. After 2.5 min, the precolumn cartridge was connected to the separating PepMap C18 column (stationary phase: C18 PepMap 100, 3 µm; column: 75 µm i.d., 150 mm; Dionex Corp.). Buffers were 0.1% HCOOH, 3% ACN (A) and 0.1% HCOOH and 95% ACN (B). The peptide separation was achieved with a linear gradient from 5 to 30% B for 25 min at 200 nL/min^-1^. Including the regeneration step at 100% B and the equilibration step at 100% A, one run took 45 min. Eluted peptides were analysed on-line with a LTQ XL ion trap (Thermo Electron Corp.) using a nano-electrospray interface. Ionization (1.5 kV ionization potential) was performed with liquid junction and a noncoated capillary probe (10 mm i.d.; New Objective Inc., NJ). Peptide ions were analysed using Xcalibur 2.07 with the following data-dependent acquisition steps: (1) full MS scan (mass-to-charge ratio (m/z) 400 to 1900, centroid mode), (2) MS2 (qz = 0.25, activation time = 30 ms, and collision energy = 35%; centroid mode). Steps 2 was repeated for the three major ions detected in step 1. Step 3 was performed on a neutral loss of H_3_PO_4_ in the MS2 spectra. Dynamic exclusion was set to 30 sec.

Database search was performed with Bioworks 3.3.1 (Thermo Electron Corp.). Enzymatic cleavage was declared as no enzyme (maximum peptide length of 40 aa). Cys carboxyamidomethylation was set to static modifications. Met oxidation and phosporylation of Ser, Thr and Tyr were used for possible modification for MS spectra. Precursor mass and fragment mass tolerance were 2.0 and 0.8, respectively. A personnal database containing ABF3, keratins and proteases sequences was used. Identification of phosphorylation sites were ﬁltered according (1) to their cross-correlation score (Xcorr), superior to 1.7, 2.2, and 3.3 for mono-, di-, and tricharged peptides, respectively, (2) to their probability inferior to 0.05 and (3) to the visually confirmation of similarity between the experimental and the theoretical MS2 spectra.

***Quantitative analysis of gene expression***

Total RNAs were extracted from epidermal fragments enriched in guard cell prepared essentially as described [1]. Four- to six-weeks-old leaves were blended 2 times for 1 min in ice-cold deionised water. Leaf fragments were recovered on a 200 μm mesh, extensively washed with deionised water and treated for 2 h in enzyme solution 1. Epidermal fragments were extensively rinsed with water, resuspended in 0.5X MS liquid medium. After a 1 h resting period, epidermal peals were treated by 50 μM ABA or ethanol for 3 h and then rapidly vacuumed dried and frozen in liquid nitrogen before RNA extraction. DNA-free RNAs were purified using the RNeasy plant mini kit according to the manufacturer’s instructions (Qiagen). First-strand cDNA was synthesized from 200 ng to 500 ng of total RNA using the Superscript III first-strand synthesis system (Invitrogen). The sequence of the primers used in quantitative RT-PCR experiments (qRT-PCR) to amplify *ABF1* (*At1g49720*), *ABF2* (*At1g45249*), *ABF3* (*At4g34000*), *ABF4* (*At3g19290*), *At2g36640*, *ACTIN2* (*At3g18780*) and *TIP41-like* gene (*At4g34270*) are given in the following table. qRT-PCR reactions were performed on a Roche LightCycler with the LightCycler FastStart DNA Masterplus SYBR Green I kit (Roche) using the following cycling sequence: [1 cycle (95°C, 10 min); 46 cycles (95°C, 5 sec; 60°C, 5 sec, 72°C, 15 sec)]. In these conditions, the amplification efficiency of all primer combinations was >86%. For gene expression analysis at least two independent biological replicates and two technical replicates using two independent cDNA syntheses from the same RNA sample were used. Gene expression was normalized to *ACTIN2* and *TIP41-like* gene expression used as constitutive controls.

***List of primers used in this study***

| **Name (Use)** | **Sequence** |
| --- | --- |
| ABF3_F (cloning) | AAAACTCGAGGGGTCTAGATTAAACTTCAAGAG |
| ABF3_R (cloning) | AAAACTCGAGCTAAGATGCATCCAGGGCGCTCTTTGG |
| NcoIYFP_F (cloning) | AAACCATGGGCAAGGGCGAGG |
| NcoIOST1_R (cloning) | TTTCCATGGTCACATTGCGTACAC |
| attB1ABF3_F  (Gateway recombination) | GGGGACAAGTTTGTACAAAAAAGCAGGCTTGATGGGGTCTAGATTAAACTT |
| attB2ABF3_R  (Gateway recombination) | GGGGACCACTTTGTACAAGAAAGCTGGGTGCTACCAGGGACCCGTCAA |
| *att*B2ABF3TA_R  (Gateway recombination) | GGGGACCACTTTGTACAAGAAAGCTGGGTGCTACCAGGGACCCGCCAATGTCCTTCGCAAGC |
| *att*B1OST1_F  (Gateway recombination) | GGGGACAAGTTTGTACAAAAAAGCAGGCTTGATGGATCGACCAGCAGTGAGT |
| *att*B2OST1_R  (Gateway recombination) | GGGGACCACTTTGTACAAGAAAGCTGGGTGTCACATTGCGTACACAATCTC |
| ABF3S32_F  (point-mutation) | CCATTGACTAGGCAGAACGCTGTGTTCTCGTTAACC |
| ABF3S32_R  (point-mutation) | GGTTAACGAGAACACAGCGTTCTGCCTAGTCAATGG |
| ABF3S126_F  (point-mutation) | GTTTGCAGAGACAAGGTGCACTTACCTTGCCTCGGACG |
| ABF3S126_R  (point-mutation) | CGTCCGAGGCAAGGTAAGTGCACCTTGTCTCTGCAAAC |
| ABF3S134_F  (point-mutation) | CCTTGCCTCGGACGATTGCTCAGAAAAGGGTTGATGATGTC |
| ABF3S134_R  (point-mutation) | GACATCATCAACCCTTTTCTGAGCAATCGTCCGAGGCAAGG |
| qABF1_F (qRT-PCR) | CACATCAGAGACTGCCTCCA |
| qABF1_R (qRT-PCR) | AACAGTACCCCCTGCTCCTC |
| qABF2_F (qRT-PCR) | CAGGATCATCAGAATCTCTTTTGC |
| qABF2_R (qRT-PCR) | AGCGACAACGACAAACAACA |
| qABF3_F (qRT-PCR) | AACCGTTCTCAACCTGCAAC |
| qABF3_R (qRT-PCR) | TTGGAGTCAGATCAGGTGACAT |
| qABF4_F (qRT-PCR) | ACCATGGTGAAGGATGAAGC |
| qABF4_R (qRT-PCR) | AGGTCAGGGCACAAAATGTC |
| qAt2g36640_F  (qRT-PCR) | TTGGTCTCTTCGGCTTTTTG |
| qAt2g36640_R  (qRT-PCR) | GGAGGAAATGAGATTGGAAGG |
| qACTIN2_F (qRT-PCR) | GCACCCTGTTCTTCTTACCG |
| qACTIN2_R (qRT-PCR) | AACCCTCGTAGATTGGCACA |
| qAt4g34270_F (qRT‑PCR) | GTGAAAACTGTTGGAGAGAAGCAA |
| qAt4g34270 (qRT‑PCR) | TCAACTGGATACCCTTTCGCA |
