# Supplementary material for: The Arabidopsis ABA-Activated Kinase OST1 Phosphorylates the bZIP Transcription Factor ABF3 and Creates a 14-3-3 Binding Site Involved in Its Turnover
Source: PLoS One. 2010 Nov 10;5(11):e13935. doi: 10.1371/journal.pone.0013935 (PMC2978106; doi:10.1371/journal.pone.0013935)
Supplement: Table S1 — (0.14 MB DOC) [file pone.0013935.s002.doc]

***Table S1:*** *Putative OST1 substrates identified in Arabidopsis annotated protein database using the MAST program.*

| Rank | E-value^a^ | AGI | Description | Peptide  sequence | Position  p-value^b^ |
| --- | --- | --- | --- | --- | --- |
| 1 | 1,6 | At3g09070 | Glycine-rich protein | LRRTKSFSAS | 7,60E-08 |
| 2 | 1,8 | At4g40020 | Unknown protein | LVRRKSLSFS | 9,20E-08 |
| 3 | 1,9 | At1g20440 | Dehydrin COR47 | LHRSNSSSSS | 2,40E-07 |
| 4 | 2,3 | At4g27590 | Copper-binding protein | LIRTSSFTWK | 4,80E-07 |
| 5 | 2,4 | At1g20450 | Dehydrin ERD10/LTI45 | LHRSNSSSSS | 3,00E-07 |
| 5 | 2,4 | At2g38070 | Glycine-rich protein | LRRTKSFSAS | 1,20E-07 |
| 7 | 4,1 | At5g24880 | Calmodulin-binding protein-related | LSRTKSLGRK | 3,00E-07 |
|  | - |  |  | LLRRRSFDRP | 1,10E-05 |
| 8 | 4,3 | At5g01170 | Glycine-rich protein | LRRTKSFSAK | 2,40E-07 |
| 9 | 4,5 | At5g66400 | Dehydrin RAB18 | LHRSGSGSSS | 7,80E-07 |
| 10 | 4,9 | At4g19760 | Glycosyl hydrolase family 18 protein | LSRAGSFSF* | 4,30E-07 |
| 11 | 5,3 | At1g76690 | 12-oxophytodienoate reductase OPR2 | LTRQKSYGSV | 4,50E-07 |
| 12 | 6 | At2g02835 | Zinc ion binding protein | LWRANTISIV | 9,90E-07 |
| 13 | 6,4 | At2g31560 | Unknown protein | LTRAKSLTDD | 1,00E-06 |
| 14 | 8,1 | At5g61710 | Unknown protein | LRRRRTSNTR | 1,70E-06 |
| 15 | 8,8 | At1g55915 | Zinc ion binding protein | LSRQPSLSFL | 6,90E-07 |
| 16 | 8,9 | At2g21490 | Dehydrin protein | LRRSGSSSSS | 1,60E-06 |
| 17 | 9,2 | At5g59200 | Pentatricopeptide repeat-containing protein | LSRRKTLISV | 5,00E-07 |
| 18 | 11 | At3g19290 | ABRE Binding Factor ABF4 (AREB2) | LQRQGSLTLP | 8,10E-07 |
|  | - |  |  | LRRTLTGPW* | 3,10E-05 |
|  | - |  |  | LARQSSVYSL | 4,40E-05 |
| 18 | 11 | At1g45249 | ABRE Binding Factor ABF2 (AREB1) | LQRQGSLTLP | 8,70E-07 |
|  | - |  |  | LTRQGSIYSL | 5,60E-06 |
| 18 | 11 | At3g49150 | F-box family protein | LRRTLSLRSL | 5,80E-07 |
|  | - |  |  | LKRSLSSKTL | 7,10E-05 |
| 21 | 12 | At4g26950 | Unknown protein | LRRSRSSSSS | 2,70E-06 |
| 21 | 12 | At4g34000 | ABRE Binding Factor ABF3 (DPBF5) | LQRQGSLTLP | 8,80E-07 |
|  | - |  |  | LRRTLTGPW* | 3,10E-05 |
|  | - |  |  | LTRQNSVFSL | 3,10E-05 |
| 21 | 12 | At3g45243 | ECA1 gametogenesis protein | LARAPSLTLA | 3,50E-06 |
| 24 | 13 | At3g12870 | Unknown protein | LRRAKSLRVE | 2,00E-06 |
| 24 | 13 | At4g23620 | 50S ribosomal protein | LRRAKTLPKT | 1,60E-06 |
| 24 | 13 | At2g43340 | Unknown protein | LKRTKSLTDD | 2,20E-06 |
| 24 | 13 | At5g10300 | Hydrolase, alpha/beta fold protein | LHRQGSFFTE | 1,60E-06 |
| 28 | 15 | At3g50980 | Dehydrin protein | LHRSGSSSSS | 3,90E-06 |
| 29 | 17 | At3g56850 | ABRE Binding Factor AREB3 (DPBF3) | LSRQGSLTLP | 1,80E-06 |
|  | - |  |  | LNRQSSLYSL | 4,10E-05 |
|  | - |  |  | LRRTSSAPF* | 6,00E-05 |
| 29 | 17 | At2g41070 | ENHANCED EM LEVEL (DPBF4) | LVRQGSLTLP | 2,10E-06 |
|  | - |  |  | LTRQNSLYSL | 6,70E-06 |
|  | - |  |  | LRRTNSASL* | 4,30E-05 |
| 29 | 17 | At4g19970 | Unknown protein | LTRSKSISFR | 7,60E-07 |
| 29 | 17 | At4g08480 | MAPKKK9 | LLRQGSFGSV | 7,10E-07 |
| 33 | 18 | At5g20900 | Unknown protein | LNRAPSFSST | 3,10E-06 |
| 33 | 18 | At5g19520 | Ion channel domain-containing protein | LVRRKSLSRS | 7,90E-07 |
| 33 | 18 | At1g49720 | ABRE Binding Factor ABF1 | LQRQGSLTLP | 1,50E-06 |
|  | - |  |  | LARQSSLYSL | 8,80E-06 |
|  | - |  |  | LRRTLTGPW* | 4,90E-05 |
|  | - |  |  | LERQQTLGEM | 9,60E-05 |
| 36 | 20 | At5g06280 | Unknown protein | LRRTKSISNM | 4,20E-06 |
| 36 | 20 | At5g06280 | Unknown protein | LRRTKSISNM | 4,20E-06 |
| 36 | 20 | At4g19750 | Glycosyl hydrolase protein | LSRAGSFSLT | 1,80E-06 |
| 39 | 21 | At1g22110 | Unknown protein | LSRTSSSSSS | 2,30E-06 |
| 39 | 21 | At1g10070 | ATBCAT-2 | LSRAKSRGFS | 1,90E-06 |
| 41 | 23 | At5g66080 | PP2C protein | LSRASSLKTP | 1,90E-06 |
| 41 | 23 | At3g27320 | Unknown protein | LSRRNSLGSS | 1,60E-06 |
| 41 | 23 | At5g20290 | 40S ribosomal protein S8 (RPS8A) | LVRTKTLVKS | 3,40E-06 |
| 41 | 23 | At3g54320 | Transcription factor | LRRQSSGFSR | 2,10E-06 |
| 41 | 23 | At1g66890 | Similar to 50S ribosomal protein-related | LRRRKTLRLL | 4,90E-06 |
| 41 | 23 | At3g22250 | UDP-glucosyl transferase family protein | LERTKSLRWI | 1,60E-06 |
| 41 | 23 | At1g04540 | C2 domain-containing protein | LRRTKSDTSS | 1,20E-06 |
| 48 | 24 | At1g68330 | Unknown protein | LRRSSSLSSS | 2,90E-06 |
| 49 | 26 | AtMg00210 | Mitochondrial ribosomal protein L5 | LARQSTLRGH | 4,50E-06 |
| 49 | 26 | At2g07725 | 60S ribosomal protein L5 (RPL5) | LARQSTLRGH | 4,50E-06 |
| 51 | 28 | At1g10570 | Ulp1 protease family protein | LKRQRSLLTR | 1,50E-06 |
| 52 | 33 | At1g71900 | Similar to permease-related | LRRQESLRSP | 3,10E-06 |
| 52 | 33 | At3g04070 | Transcription factor | LKRQKSSCSF | 3,00E-06 |
| 52 | 33 | At5g10660 | Calmodulin-binding protein-related | LTRSKSLGRK | 2,60E-06 |
|  | - |  |  | LLRRRSFDHP | 1,10E-05 |
| 52 | 33 | At4g11910 | Unknown protein | LPRTYTLTHS | 4,00E-06 |
| 56 | 34 | At1g74870 | Protein binding / zinc ion binding | LERTSSFASS | 3,80E-06 |
| 57 | 35 | At2g02860 | Sucrose transporter 3 | LQRLPTLSSS | 2,40E-06 |
| 57 | 35 | At1g55240 | Unknown protein | LHRAKSLVNL | 3,70E-06 |
|  | - |  |  | LVRSSSIAFQ | 8,40E-05 |
| 59 | 36 | At4g17070 | Similar to Os03g0100300 | LRRCPSIKRR | 3,10E-06 |
| 60 | 37 | At4g08510 | Unknown protein | LRRSHSMTTR | 2,10E-06 |
| 61 | 38 | At1g35820 | Unknown protein | LLRTKTGITV | 4,30E-06 |
| 62 | 39 | At3g18010 | Transcription factor | LYRQGTRTPS | 3,50E-06 |
| 62 | 39 | At2g25140 | Heat shock protein | LLRSRSLSSS | 1,30E-06 |
| 62 | 39 | At5g10210 | Unknown protein | LRRRKTTPFL | 6,50E-06 |
| 65 | 41 | At4g15975 | Zinc finger protein | LSRSHSFRSP | 5,60E-06 |
| 65 | 41 | At1g55420 | Embryo sac development arrest 11 | LGRRKSFCTL | 1,80E-06 |
| 67 | 42 | At3g25160 | ER lumen protein retaining receptor protein | LTRQKTCSGL | 4,90E-06 |
| 67 | 42 | At2g36270 | DNA binding factor ABI5 | LPRQGSLTLP | 3,00E-06 |
|  | - |  |  | LGRQSSIYSL | 1,40E-05 |
| 67 | 42 | At1g08400 | Chromosome structural maintenance protein | LLRASTLTSN | 1,70E-06 |
| 70 | 43 | At1g34770 | MAGE-8 antigen-related | LQRARSSSTG | 5,90E-06 |
| 71 | 44 | At1g01780 | LIM domain-containing protein | LTRTPSKISS | 6,90E-06 |
| 71 | 44 | At4g22920 | Similar to the tomato stay-green protein 1 | LPRTYTLTHS | 5,30E-06 |
| 73 | 45 | At3g12955 | Auxin-responsive protein-related | LRRCKSVSTQ | 1,10E-05 |
| 73 | 45 | At5g22750 | DNA binding protein | LRRTKSSTDR | 1,40E-06 |
| 73 | 45 | At1g67630 | DNA polymerase alpha subunit B family | LLRQRSFYPL | 2,30E-06 |
|  | - |  |  | LKRCVTLCIN | 9,60E-05 |
| 76 | 47 | At4g39900 | Unknown protein | LQRQASLSTD | 6,20E-06 |
| 76 | 47 | At1g15580 | Transcription factor IAA5 | LERTKSSYVK | 9,50E-06 |
| 76 | 47 | At4g20450 | Leucine-rich repeat protein kinase | LTRTKSSTLP | 1,70E-06 |
| 79 | 48 | At1g05040 | UBA-like protein | LRRRYSTTSV | 9,60E-06 |
| 80 | 49 | At5g16200 | 50S ribosomal protein-related | LRRRKTLRML | 1,00E-05 |

^a^ The E-value of a sequence in a database is the expected number of sequences in a random database of the same size that would match the motif as well as the sequence does. Results are displayed for E-value ≤50

^b^ The position p-value is the probability of a single random subsequence of the length of the motif scoring at least as well as the observed match. Only peptides with position p-value ≤0.0001 are displayed.
