# Supplementary material for: The Arabidopsis ABA-Activated Kinase OST1 Phosphorylates the bZIP Transcription Factor ABF3 and Creates a 14-3-3 Binding Site Involved in Its Turnover
Source: PLoS One. 2010 Nov 10;5(11):e13935. doi: 10.1371/journal.pone.0013935 (PMC2978106; doi:10.1371/journal.pone.0013935)
Supplement: Table S2 — (0.11 MB DOC) [file pone.0013935.s003.doc]

#### Table S2: Putative OST1 substrates expressed in Arabidopsis guard cell identified using the MAST program.

| Rank | E-value^a^ | AGI | Description | Peptide  sequence | Position  p-value^b^ |
| --- | --- | --- | --- | --- | --- |
| 1 | 0.34 | At1g20440 | Dehydrin COR47 | LHRSN**S**SSSS | 2.40e-07 |
| 2 | 0.42 | At1g20450 | Dehydrin ERD10/LTI45 | LHRSN**S**SSSS | 3.00e-07 |
| 3 | 0.79 | At5g66400 | Dehydrin RAB18 | LHRSG**S**GSSS | 7.80e-07 |
| 4 | 0.94 | At1g76690 | 12-oxophytodienoate reductase OPR2 | LTRQK**S**YGSV | 4.50e-07 |
| 5 | 1.9 | At3g19290 | ABRE Binding Factor ABF4 (AREB2) | LQRQG**S**LTLP | 8.10e-07 |
|  | - |  |  | LRRTL**T**GPW* | 3.10e-05 |
|  | - |  |  | LARQS**S**VYSL | 4.40e-05 |
| 6 | 2.2 | At4g34000 | ABRE Binding Factor ABF3 (DPBF5) | LQRQG**S**LTLP | 8.80e-07 |
|  | - |  |  | LRRTL**T**GPW* | 3.10e-05 |
|  | - |  |  | LTRQN**S**VFSL | 3.10e-05 |
| 7 | 2.3 | At1g76180 | Dehydrin ERD14 | LHRSD**S**SSSS | 2.30e-06 |
| 8 | 3.1 | At5g20900 | Jasmonate-ZIM-domain protein 12, JAZ12 | LNRAP**S**FSST | 3.10e-06 |
| 9 | 4.1 | At5g20290 | 40S ribosomal protein S8 | LVRTK**T**LVKS | 3.40e-06 |
| 10 | 4.5 | At2g07725 | 60S ribosomal protein L5 | LARQS**T**LRGH | 4.50e-06 |
| 11 | 7.8 | At4g22920 | Similar to tomato stay-green protein 1 | LPRTY**T**LTHS | 5.30e-06 |
| 12 | 8.3 | At4g39900 | Unknown protein | LQRQA**S**LSTD | 6.20e-06 |
| 12 | 8.3 | At1g69250 | Nuclear transport protein | LARQN**T**LVVL | 3.50e-06 |
| 14 | 9 | At1g71710 | Inositol polyphosphate 5-phosphatase | LDRQP**S**IKTV | 2.50e-06 |
|  | - |  |  | LQRAL**T**FTDA | 2.70e-05 |
|  | - |  |  | LRRRN**S**ETLR | 3.90e-05 |
| 15 | 11 | At1g47420 | Unknown protein | LSRHR**T**LITV | 7.50e-06 |
| 16 | 14 | At5g66510 | Gamma carbonic anhydrase 3 | LVRQN**T**RIPS | 9.80e-06 |
|  | - |  |  | LSRHR**T**LMNV | 3.40e-05 |
| 16 | 14 | At4g17940 | Unknown protein | LMRTG**S**MPVL | 9.70e-06 |
| 18 | 15 | At1g64970 | Gamma tocopherol methyltransferase | LFRSP**S**SSSS | 7.80e-06 |
| 18 | 15 | At2g38780 | Unknown protein | LFRTR**S**NSSS | 5.90e-06 |
| 18 | 15 | At1g19580 | Gamma carbonic anhydrase 1 | LVRQN**T**RIPS | 1.00e-05 |
|  | - |  |  | LSRHR**T**LMNV | 3.60e-05 |
| 21 | 18 | At2g34690 | Accelerated cell death 11 | LVRAS**S**SITR | 1.60e-05 |
| 22 | 19 | At5g08280 | Hydroxymethybilane synthase | LTRQP**S**SRVN | 8.90e-06 |
| 22 | 19 | At1g09140 | RNA binding protein | LSRSR**S**LYSS | 1.30e-05 |
| 24 | 20 | At2g47970 | NPL4 family protein | LDRAK**S**LPLV | 8.90e-06 |
| 24 | 20 | At5g58440 | PHOX (PX) domain-containing protein | LSRSP**S**SSSS | 6.30e-06 |
| 26 | 21 | At2g41680 | NADPH-dependent thioredoxin reductase | LLRQP**T**RTRS | 7.00e-06 |
| 26 | 21 | At4g38970 | Fructose-biphosphate aldolase | LFRQP**S**SASV | 9.50e-06 |
| 28 | 24 | At5g19760 | Dicarboxylate / Tricarboxylate Carrier (DTC) | LLRQA**T**YTTA | 1.50e-05 |
| 29 | 25 | At4g31390 | ABC1 protein | LRRSR**T**FSAV | 6.70e-06 |
| 30 | 28 | At5g67180 | AP2 domain-containing transcription factor | LRRQS**T**GFPR | 1.40e-05 |
| 31 | 29 | At5g48380 | Leucine-rich repeat protein / protein kinase | LGRLK**T**FSVS | 8.30e-06 |
| 31 | 29 | At2g20670 | Unknown protein | LYRTS**S**IETK | 8.10e-06 |
| 31 | 29 | At1g19660 | Wound-responsive protein | LRRYK**S**IKCL | 1.60e-05 |
| 34 | 31 | At4g13890 | Serine hydroxymethyltransferase 5 | LHRAV**T**ITLD | 1.20e-05 |
|  | - |  |  | LCRSR**S**LEAF | 7.90e-05 |
| 34 | 31 | At2g43350 | Glutathione peroxidase 3 | LYRYP**S**SPST | 2.80e-05 |
| 36 | 33 | At5g50240 | Protein-l-isoaspartate methyltransferase 2 | LHRYN**S**SSSS | 2.00e-05 |
| 36 | 33 | At5g17310 | UTP-glucose-1-phosphate uridylyltransferase | LSRFK**S**IPSI | 1.50e-05 |
| 38 | 35 | At5g40890 | Chloride channel A | LKRHR**T**LSST | 8.20e-06 |
| 39 | 36 | At2g39690 | Unknown protein | LSRRP**S**SCSR | 1.30e-05 |
| 39 | 36 | At1g10960 | Ferredoxin 1 | LRRQQ**T**PISL | 4.60e-05 |
| 41 | 38 | At2g42280 | Basic helix-loop-helix protein | LRRHC**S**LSSR | 1.90e-05 |
| 42 | 39 | At1g07650 | Leucine-rich repeat transmembrane kinase | LSRSL**S**FSTS | 6.90e-06 |
| 42 | 39 | At2g31200 | Actin depolymerizing factor 6 | LQRKK**T**HRYV | 5.10e-05 |
| 44 | 40 | At4g11600 | Glutathione peroxidase 6 | LLRSL**S**SSSS | 3.20e-05 |
| 44 | 40 | At5g19550 | Aspartate aminotransferase 2 | LMRSK**S**LLPF | 1.80e-05 |
| 46 | 41 | At5g41670 | 6-phosphogluconate dehydrogenase protein | LLRAK**S**LEKG | 1.50e-05 |
| 47 | 42 | At2g19450 | Triacylglycerol biosynthesis defect 1 | LRRRK**S**RSDS | 1.50e-05 |
| 48 | 43 | At5g10470 | Kinesin motor protein-related | LLRRN**S**ISTP | 6.00e-06 |
| 48 | 43 | At3g02360 | 6-phosphogluconate dehydrogenase protein | LIRAK**S**IEKG | 1.60e-05 |
| 48 | 43 | At1g16080 | Unknown protein | LRRSE**S**LKPS | 2.50e-05 |
| 51 | 44 | At1g47260 | Carbonate dehydratase | LSRHR**T**LMNV | 2.90e-05 |
| 51 | 44 | At5g51110 | Similar to dehydratase family | LLRQP**S**RSIL | 3.70e-05 |
| 53 | 45 | At1g14730 | Similar to Arabidopsis cytochrome b561 | LKRHS**S**LSTL | 3.70e-05 |
| 54 | 47 | At4g19170 | 9-cis-epoxycarotenoid dioxygenase 4 | LLRRR**S**SSPT | 1.40e-05 |
| 55 | 49 | At5g03900 | Identical to protein At5g03900 precursor | LQRTA**S**GSSR | 2.10e-05 |
| 56 | 50 | At5g19500 | Tryptophan/tyrosine permease protein | LLRLP**T**FSVP | 1.80e-05 |

^a^ The E-value of a sequence in a database is the expected number of sequences in a random database of the same size that would match the motif as well as the sequence does. Results are displayed for E-value ≤50.

^b^ The position p-value is the probability of a single random subsequence of the length of the motif scoring at least as well as the observed match. Only peptides with position P-value ≤0.0001 are displayed.
