# Supplementary figures and images for: The Arabidopsis ABA-Activated Kinase OST1 Phosphorylates the bZIP Transcription Factor ABF3 and Creates a 14-3-3 Binding Site Involved in Its Turnover
Source: PLoS One. 2010 Nov 10;5(11):e13935. doi: 10.1371/journal.pone.0013935 (PMC2978106; doi:10.1371/journal.pone.0013935)

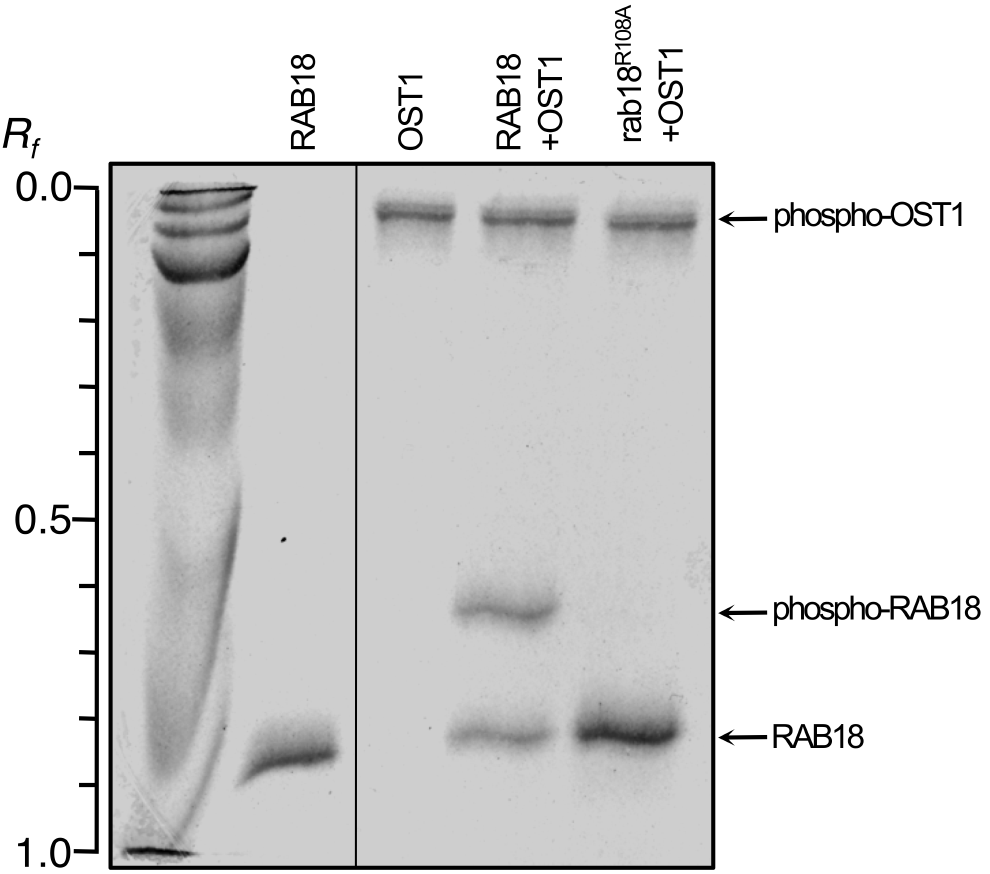

Supplement: Figure S1 — OST1 phosphorylates RAB18 in vitro. (10xHis-)RAB18 and the Arg108 to Ala mutant (10xHis-)rab18R108A was phosphorylated by OST1 in vitro. Protein were analyzed in a SDS-PAGE gel containing Mn2+-Phos-tag and stained with Coomassie blue. Phos-tag specifically chelates phosphate group and slow down the migration of phosphorylated protein [75]. (0.17 MB TIF) [file pone.0013935.s004.tif]

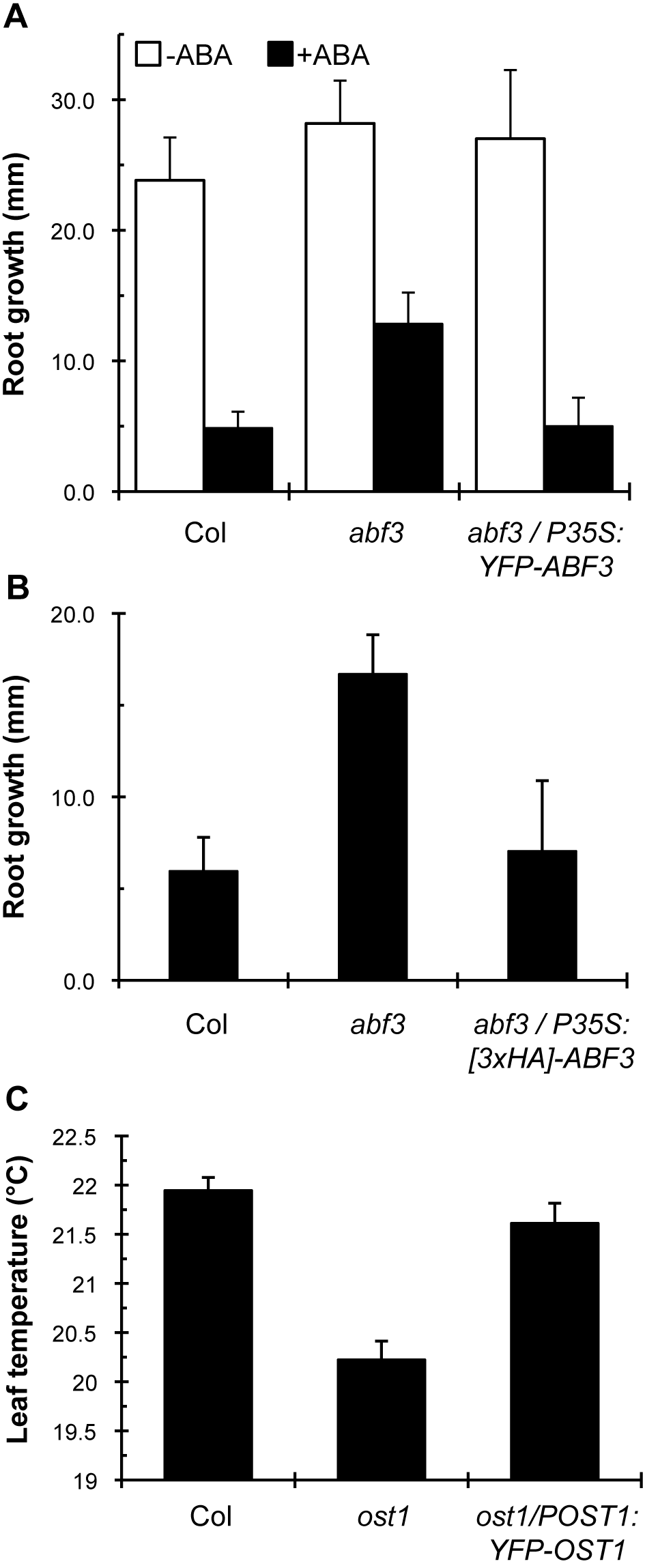

Supplement: Figure S2 — Complementation of abf3 and ost1 mutants by expression of fusion proteins. (A) The root growth of Arabidopsis WT (Col), abf3 mutant and abf3 transgenic line expressing YFP-ABF3 under the control of the 35S promoter was measured in absence and presence of 30 µM ABA [76]. (B) The root growth of Arabidopsis WT (Col), abf3 and abf3 transgenic line expressing [3xHA]-ABF3 under the control of the 35S promoter was measured in presence of 30 µM ABA. (C) Detached leaf temperature analysis of Arabidopsis WT (Col), ost1 (srk2e) and ost1 transgenic line expressing YFP-OST1 under the control of the OST1 promoter. In these analyses, error bars represent the standard deviation of the mean. (0.09 MB TIF) [file pone.0013935.s005.tif]

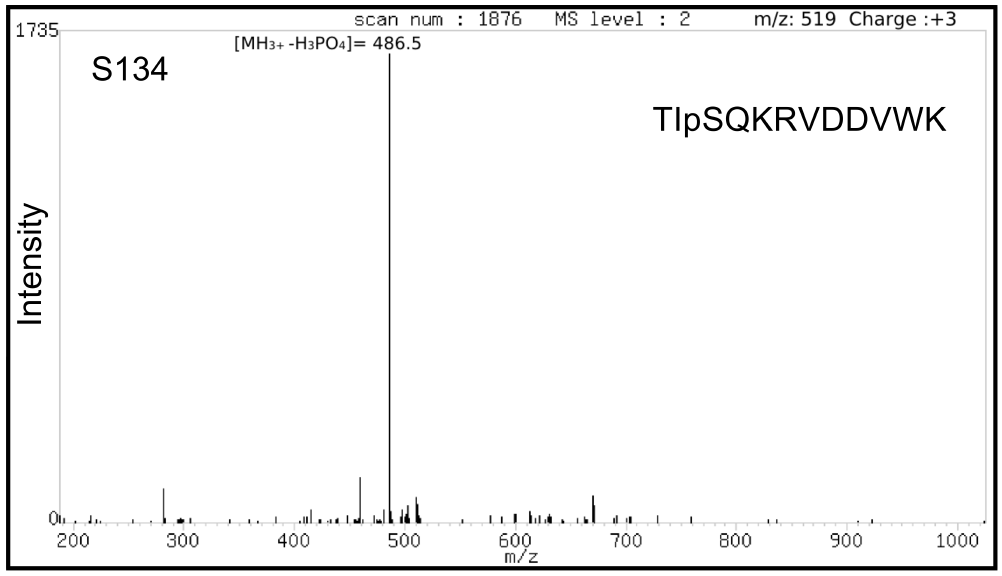

Supplement: Figure S3 — Identification of phosphorylated ABF3 S134 by LC-MS/MS. Phosphorylation of S134 is revealed in MS2 spectra by the neutral loss of phosphoric acid group (H3PO4, 98 Da) from the triply charged precursor ion TIpS134QKRVDDVWK ion at m/z 519.00 produced by trypsin hydrolysis with miscleavages. The fragmentation of this peptide was not annotated by the Bioworks 3.3.1 program. (0.03 MB TIF) [file pone.0013935.s006.tif]
